# PRMT1 in human neoplasm: cancer biology and potential therapeutic target

**DOI:** 10.1186/s12964-024-01506-z

**Published:** 2024-02-08

**Authors:** Shiquan Shen, Honglong Zhou, Zongyu Xiao, Shaofen Zhan, Yonghua Tuo, Danmin Chen, Xiao Pang, Yezhong Wang, Ji Wang

**Affiliations:** 1 Department of Neurosurgery, Institute of Neuroscience, The Second Affiliated Hospital of Guangzhou Medical University, Guangzhou, 510260 China; 2 Department of Neurosurgery, The Second Affiliated Hospital of Nanchang University, Nanchang, 330006 China; 3 Department of Neurosurgery, Dushu Lake Hospital Affiliated to Soochow University, Suzhou, 215124 China; 4 Department of Neurology, Guangdong Second Provincial General Hospital, Southern Medical University, Guangzhou, 510317 China

**Keywords:** Arginine methylation, PRMT1, Cancer biology, PRMT1 inhibitor, Target

## Abstract

Protein arginine methyltransferase 1 (PRMT1), the predominant type I protein arginine methyltransferase, plays a crucial role in normal biological functions by catalyzing the methylation of arginine side chains, specifically monomethylarginine (MMA) and asymmetric dimethylarginine (ADMA), within proteins. Recent investigations have unveiled an association between dysregulated PRMT1 expression and the initiation and progression of tumors, significantly impacting patient prognosis, attributed to PRMT1’s involvement in regulating various facets of tumor cell biology, including DNA damage repair, transcriptional and translational regulation, as well as signal transduction. In this review, we present an overview of recent advancements in PRMT1 research across different tumor types, with a specific focus on its contributions to tumor cell proliferation, metastasis, invasion, and drug resistance. Additionally, we expound on the dynamic functions of PRMT1 during distinct stages of cancer progression, elucidating its unique regulatory mechanisms within the same signaling pathway and distinguishing between its promotive and inhibitory effects. Importantly, we sought to provide a comprehensive summary and analysis of recent research progress on PRMT1 in tumors, contributing to a deeper understanding of its role in tumorigenesis, development, and potential treatment strategies.

## Introduction

Cancer poses a significant threat to human health globally, given its high prevalence and complex nature. Despite substantial progress in understanding and treating tumors over the past few decades, eight key indicators of human tumors have been identified, encompassing sustained proliferation ability, evasion of growth inhibitory factors, resistance to death, replication immortality, angiogenesis induction, activation of invasion and metastasis, reprogramming of energy metabolism, and evasion of immune damage [1]. However, challenges persist in tumor treatment, including drug resistance, recurrence, and metastasis. Hence, the discovery of new therapeutic targets and strategies remains crucial to enhance the efficacy of tumor treatment.

As a pivotal post-translational modification enzyme, PRMT1 plays a crucial role in the occurrence and development of tumors by catalyzing protein arginine methylation modifications. Its impact spans various cellular processes, including gene expression regulation, cell cycle regulation, and DNA repair, significantly influencing tumor cell proliferation, invasion, metastasis, and antiapoptotic abilities [2]. The intricate involvement of PRMT1 in these processes underscores its significance in understanding the fundamental mechanisms of cancer onset and advancement, as well as its potential in predicting prognosis. Therefore, the study of PRMT1 in tumors holds both theoretical and practical importance. This review aims to comprehensively examine recent research progress on PRMT1 in tumors, summarizing its potential applications in tumor treatment. By exploring the function and regulatory mechanisms of PRMT1, we sought to deepen our understanding of the mechanisms underlying tumor occurrence, identify new therapeutic targets and strategies, and enhance the efficacy of tumor treatment. The review will be organized into three sections: first, an introduction to the structure and function of PRMT1; second, a review of the expression and function of PRMT1 in different types of tumors; and finally, a summary of the potential application value of PRMT1 as a therapeutic target, outlining future research directions.

### Structure and function of PRMT1

PRMTs play a pivotal role in transferring methyl groups from S-Adenosyl methionine (SAM) to the guanidine nitrogen of protein arginine, resulting in the production of methyl arginine and SAH (S-adenosylhomocysteine) [3]. There are nine PRMT members in total, categorized into three types. Type I PRMTs (PRMT-1, 2, 3, 4, 6, and 8) catalyze the formation of MMA and ADMA. Type II PRMTs (PRMT 5 and 9) generate MMA and SDMA. Lastly, Type III PRMTs (PRMT7) exclusively produce MMA [4]. Additionally, a Type IV PRMT produces d-NG-monomethylarginine in fungi [5].

**Fig. 1:**
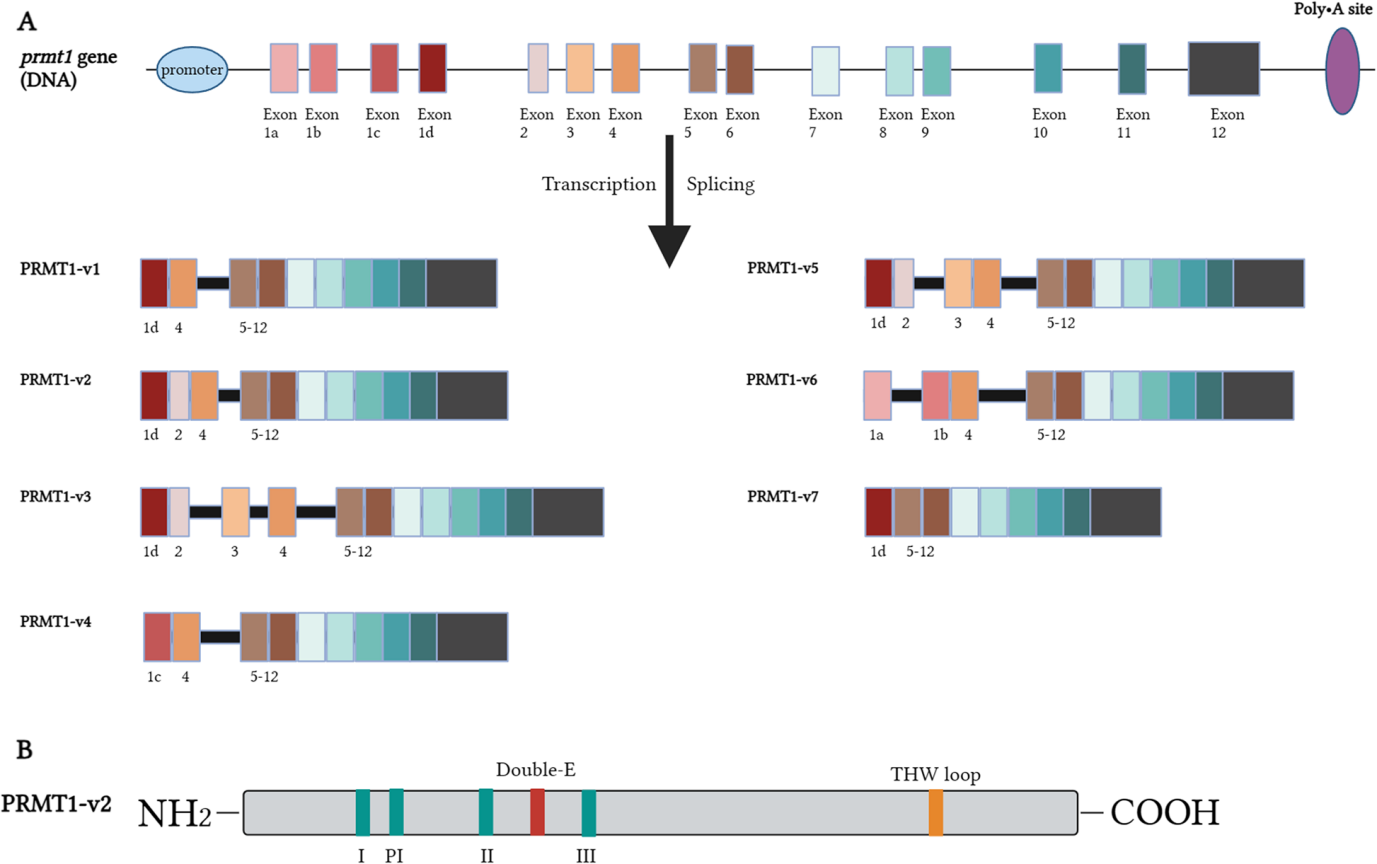
The genomic and protein structure of PRMT1. **A** The genomic structure of the *PRMT1* gene consists of 12 constitutive exons, with exon 1 further divided into 4 alternative exons. The *PRMT1* gene generates PRMT1 isoforms (v1 to v7) through transcription and splicing, along with the exon composition of these isoforms. Intron boundaries sequences are represented by the black boxes. **B** The protein structure of PRMT1-v2, which is the signature motifs (I, Post-I (PI), II, III), double E motif, and THW loop (adapted from [6])

The *PRMT1* gene is situated on human chromosome 19, precisely at 19q13.32. Comprising 12 exons and 11 introns, this gene is present in all eukaryotes and displays a high degree of conservation [6]. The homology among mammals, zebrafish, and Xenopus is over 90%, while the homology between humans and *Saccharomyces cerevisiae* is approximately 50% [7]. The complex genomic structure at the 5′ terminus of the PRMT1 gene results in the expression of seven PRMT1 isoforms, denoted as PRMT1v1-v7 (Fig. 1A). These isoforms vary in different aspects, such as molecular weight, N-terminal structure, substrate specificity, tissue specificity, subcellular localization, and tissue expression. Biochemical characterization has revealed that all isoforms, except PRMT1v7, are active [8]. Notably, the PRMT1v2 isoform contains amino acids that regulate its cytoplasmic localization and significantly contribute to the survival and invasion of breast cancer cells [9].

PRMT1, the earliest discovered protein arginine methyltransferase and the predominant Type I PRMT in mammalian cells, accounts for 85% of cellular PRMT activity [10]. Over the years, the structure of PRMT1 has undergone extensive study and characterization, consisting of two domains: the N-terminal AdoMet-binding domain and the C-terminal β-barrel domain. The N-terminal end of the AdoMet-binding domain contains a dynamic α-helix, which plays a critical role in AdoMet binding, and the active site pocket, where the methyltransfer reaction occurs, is located between these two domains [11]. Mutagenesis studies have underscored the essential role of two active site glutamates in the enzymatic activity of PRMT1. Furthermore, the α helical dimerization arm, which is responsible for PRMT1 dimerization, extends from the N-terminal of the β barrel domain and connects to the N-terminal of the AdoMet-binding domain [11]. This creates the core form of PRMT1, which is a doughnut-shaped homodimer arranged in a head-to-tail pattern that is imperative for its binding to the co-factor S-Adenosyl methionine [12]. The catalytic core of PRMT1, comprising approximately 310 highly conserved amino acids, is responsible for its methyltransferase activity. This catalytic core consists of six highly conserved peptide motifs: motif I (VLDVGSGTG), motif II (VDI), motif III (LAPDG), post-motif I (VIGIE), double E motif (SEWMGYCLFYESM), and THW loop (YTHWK), which are essential for methyltransferase activity of PRMT1 (Fig. 1B). The site that binds AdoMet is delimited by motif I, which is stabilized by motifs II and III. The glutamic acid residue of the post-motif I forms hydrogen bonds with AdoMet, which is crucial for AdoMet binding in this pocket. The substrate pocket for arginine residues is defined collectively by the double E motif, which is composed of two negatively charged glutamic acid residues (E144 and E153) that can neutralize the positively charged guanidium group of the target arginine, and the THW loop, which stabilizes the three dynamic α-helices (αX, αY, αZ) located at the N-terminus of the Rossmann fold and participates in the recognition of arginine residues [6].

PRMT1 is a pivotal enzyme involved in various cellular processes. Extensive research has revealed that PRMT1 primarily targets substrates containing conserved glycine- and arginine-rich (GAR) motifs, typically located at the C-terminus of proteins. However, Coactivator-associated arginine methyltransferase 1 (CARM1), a notable exception, predominantly methylates patterns rich in PGM motifs situated at the N-terminus or intermediate region of proteins [13]. The arginine methylation of the GAR motif by PRMT1 is particularly vital for regulating the DNA damage response of 53BP1 [14], evidenced by the resistance to γ-IR sensitivity observed in mice with lysine substituted for arginine in the MRE11 GAR motif [15]. Certain proteins that contain RGG motifs, such as FUS and nucleolin, can be specific targets for PRMT1 [16, 17]. Recent research has shown that PRMT1 mediates arginine methylation within the C-terminal RGG repeats of METTL14, thereby regulating m6A modification and promoting tumorigenesis [18]. Importantly, PRMT1 recognizes not only RGG and GAR sequences but also other amino acid sequences, suggesting that the characteristics of these substrate sequences may influence the substrate selectivity and functional regulation of PRMT1 [19].

It is now understood that in normal cells, PRMT1 plays a critical role in various physiological processes [20]. *PRMT1* deletion in mice leads to embryonic lethality, highlighting its indispensable nature for survival until birth [21]. Furthermore, PRMT1-mediated histone H4R3me2a methylation is crucial for regulating cell proliferation in various organs and serves as a key regulator of normal vertebrate development and growth [22]. Studies have demonstrated PRMT1’s involvement in regulating the self-renewal of hematopoietic stem cells and normal hematopoiesis [23], as well as in the normal development of lymphocytes [24, 25], the differentiation and proliferation of intestinal cells [26], myelin regeneration [27], and spermatogenesis [28]. Notably, the absence of *PRMT1* in mice results in a significant decrease in the proliferation of palate mesenchymal cells, ultimately preventing the palatal shelves from reaching the midline and resulting in a complete cleft palate phenotype [29]. Additionally, the PRMT1-P53 pathway plays a crucial role in the formation of epicardium-derived mesenchymal lineages during cardiac development, supporting ventricular morphogenesis and coronary artery formation [30]. In the context of vascular health, transcriptome data of abdominal aortic aneurysm patients and elderly aortas have shown decreased expression of PRMT1, revealing its role in the necessary epigenetic modification of chromatin to maintain vascular smooth muscle contraction [31]. Mice with *PRMT1* knockout exhibit characteristics of heart failure, highlighting the importance of PRMT1 in regulating alternative splicing in vivo and maintaining cardiac homeostasis [32]. Moreover, PRMT1’s modulation of cardiac I_Ks_ activity may be a crucial target for preventing excessive prolongation of action potential duration and arrhythmias in heart failure patients [33]. PRMT1 is also essential for the maintenance of normal white adipose tissue function under diet-induced obesity conditions, preventing lipid accumulation in peripheral tissues and contributing to the regulation of metabolic homeostasis [34]. Moreover, PRMT1 reduces high-fat diet-induced hepatic steatosis by increasing PGC-1α expression through the recruitment of HNF-4α to the PGC-1α promoter, thereby promoting fatty acid oxidation [35]. However, the dysregulation of PRMT1 expression has been linked to numerous diseases, with its overexpression identified as a significant factor in the development of allergic rhinitis by increasing the levels of epithelial-derived cytokines [36]. Targeting PRMT1 has also shown promise in improving patient outcomes in neurodegenerative diseases and diabetic nephropathy [37, 38]. PRMT1 overexpression has been associated with tumor progression, making it a significant target for therapeutic intervention [6]. In conclusion, PRMT1 plays an essential role in maintaining normal physiological function and, due to its diverse roles in various pathological processes, has potential as an important therapeutic target in a wide range of diseases.

### The roles of PRMT1 in cancers

The extensive involvement of PRMT1 in the development and progression of cancer has spurred extensive research to unravel its functions and regulatory mechanisms. The ultimate goal is to unveil groundbreaking opportunities and strategies for cancer treatment. In the subsequent section, we present a comprehensive review of research findings on PRMT1 in tumors, exploring its potential value in tumor therapy.

### Breast cancer

Breast cancer incidence rates have consistently risen by approximately 0.5% per year, establishing it as the predominant cancer type among women [39]. Investigations conducted on mouse models have unveiled the significant role of PRMT1 overexpression in the mammary gland in tumorigenesis, fostering PI3K-AKT pathway activity [40]. Recent research has demonstrated heightened PRMT1 expression in breast cancer specimens compared to normal tissues, establishing a correlation between elevated PRMT1 levels and malignancy in breast cancer patients [41, 42]. Moreover, escalated PRMT1 levels have been linked to unfavorable clinical outcomes in these patients [43]. Within the breast cancer microenvironment, tumor-associated macrophages (TAMs) secrete the IL-6 cytokine, which triggers PRMT1 to mediate the formation of asymmetric dimethylation of EZH2 at arginine 342 [44]. This process reinforces EZH2 stability by hindering EZH2 phosphorylation through both CDK1-mediated and AMPK-mediated pathways, thus impeding EZH2 degradation via the TRAF6 ubiquitin-proteasome pathway [43]. Additionally, PRMT1-mediated EZH2 methylation (R342) associates with SUZ12, enhancing PRC2 assembly. This, in turn, results in the suppression of P16 and P21 transcription due to elevated EZH2 expression and increased H3K27me3 enrichment at their respective promoters. Consequently, elevated EZH2 levels promote metastasis of breast cancer cells by suppressing the expression of target genes such as E-cadherin, DAB2IP, and CSTA. The diminished levels of P16 and P21 in this context accelerate cell cycle progression and cellular proliferation [45].

In breast cancer cells, PRMT1 plays a crucial role in mediating the asymmetric dimethylation of histone H4 at arginine 3 within the ZEB1 promoter, facilitating the transcriptional expression of ZEB1, a key factor in the EMT process [46]. The maintenance of proliferative signaling and the disruption of negative feedback mechanisms are integral to tumorigenesis. For instance, in triple-negative breast cancer, PRMT1 regulates the EGFR and Wnt signaling pathways, thus sustaining proliferative signaling [42, 47]. Additionally, PRMT1 reverses the repression of cyclin D1 transcription mediated by C/EBPα through the inhibition of HDAC3 corepressor activity via the weakened interaction between HDAC3 and unmethylated C/EBPα, achieved through the methylation of three arginine residues (R35, R156, and R165) on C/EBPα [41].

**Fig. 2:**
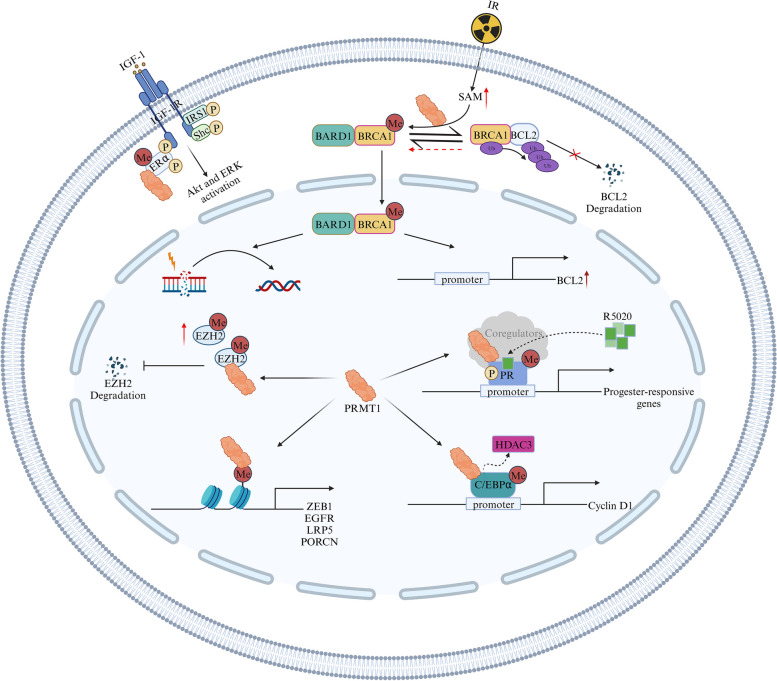
The mechanisms of action of PRMT1 in breast cancer. PRMT1 is involved in regulating multiple pathways and processes in breast cancer, including DNA damage repair, transcription, and signal transduction

Under hypoxic conditions, PRMT1 upregulates the expression of circTBC1D14 and methylates FUS, forming a complex with circTBC1D14. This complex helps maintain a balance between stress granule (SG) formation and autophagy, thus preserving cellular homeostasis [48]. PRMT1 also inhibits the transcriptional activity of P53 through multi-region methylation, preventing breast cancer cells from undergoing apoptosis or senescence [49]. Radiation-induced damage to breast cancer cells increases levels of S-Adenosyl methionine and enhances PRMT1 enzymatic activity, leading to the methylation of BRCA1. This methylated BRCA1, facilitated by BARD1, translocates to the nucleus and upregulates the antiapoptotic protein BCL2 [50]. Furthermore, PRMT1 is involved in DNA homologous recombination repair, a process regulated by the PRMT1/c-Myc network [51]. These mechanisms collectively contribute to the epigenetic defense of breast cancer cells against ionizing radiation, conferring resistance to olaparib, a PARP inhibitor. As a co-factor, PRMT1 forms a repressive multiprotein complex with HP1γ, HDAC1/2, and LSD1. This complex anchors the PR promoter, inducing local chromatin closure and maintaining the silent state of PR target genes in the absence of progesterone [52]. Upon exposure to progesterone, PRMT1 acts as a coactivator by methylating PR at arginine 637. This process promotes the expression of genes involved in cell proliferation and metastasis by dissociating the repressive multiprotein complex. However, the stability of R637me-PR diminishes, resulting in PR degradation, intensifying the PR cycle, and ultimately accelerating PR transcriptional activity [53]. Moreover, PRMT1 effectively methylates ERα when triggered by IGF-1, which enables ERα to bind with IGF-1R. Upon binding, IGF-1R phosphorylates ERα on residue Y219, strengthening their interaction. Notably, IGF-1R also phosphorylates tyrosine residues of IRS1 and Shc, creating docking platforms for PI3K and Grb2, which activate the Akt and ERK pathways, respectively [54]. In conclusion, recent insights into the multifaceted role of PRMT1 in breast cancer underscore the need for further investigation, positioning PRMT1 as a promising therapeutic target in breast cancer treatment. The complexity of its involvement in various pathways suggests its potential as a focal point for therapeutic intervention in breast cancer (Fig. 2).

### Pancreatic cancer

**Fig. 3:**
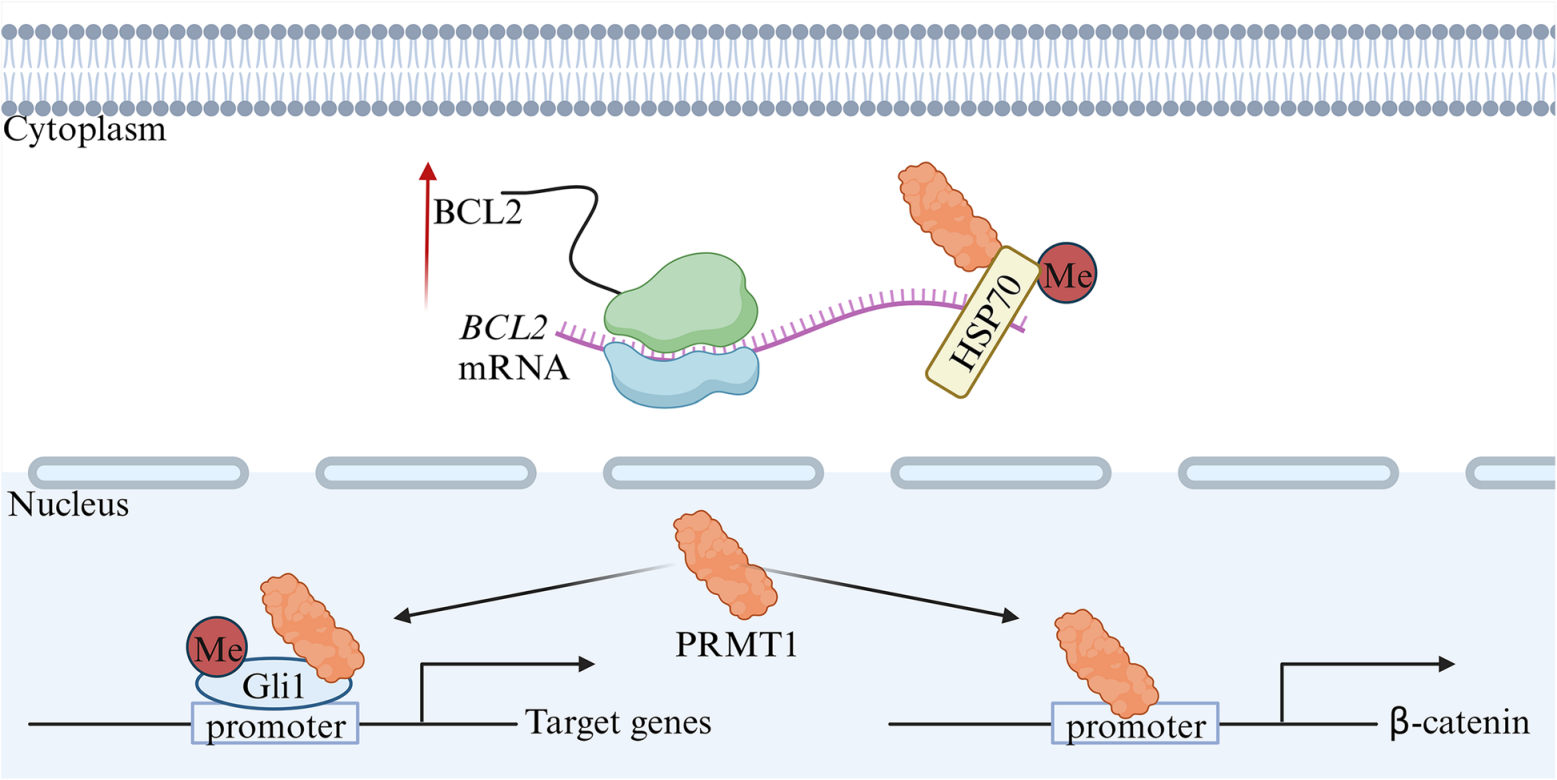
The mechanisms of action of PRMT1 in pancreatic cancer involve transcription and interpretation

By mediating ADMA of substrate proteins, the post-translational modification enzyme PRMT1 plays a significant role in pancreatic cancer (Fig. 3). Recent research conducted by Virginia et al. has illuminated the importance of PRMT1-generated ADMA in regulating RNA processing and controlling gene expression associated with DNA damage response (DDR). Such regulation is critical for maintaining genome stability and promoting tumor growth [55]. Previous studies have established that the overactivation of β-catenin/TCF signaling contributes to the growth, migration, and metastasis of pancreatic cancer cells. Notably, PRMT1 plays a pivotal role in this context by facilitating the expression of the β-catenin protein through its binding to the CTNNB1 promoter region [56]. Additionally, PRMT1 enhances the oncogenic function of Gli1, a transcription factor, by methylating it at the R597 site. This methylation event promotes the accumulation of Gli1 on its target gene promoter, enhancing its transcriptional activity and subsequently increasing the expression of target genes [57]. Another noteworthy discovery is the PRMT1-HSP70-BCL2 pathway, which has been associated with drug resistance in cancer cells, including resistance to the chemotherapy drug gemcitabine. The mechanism underlying this pathway involves PRMT1 methylation of HSP70 at the R416 and R447 sites. This methylation event enhances the binding and stabilization of HSP70 with *BCL2* mRNA, leading to increased BCL2 protein expression. The accumulation of BCL2, a crucial antiapoptotic protein, can benefit pancreatic cancer cells as it protects them from apoptosis induced by cellular stresses and therapeutic interventions [58]. These findings offer valuable insights into the intricate mechanisms underlying pancreatic cancer development and underscore the potential role of PRMT1 as a therapeutic target in combating this formidable disease.

### Lung cancer

**Fig. 4:**
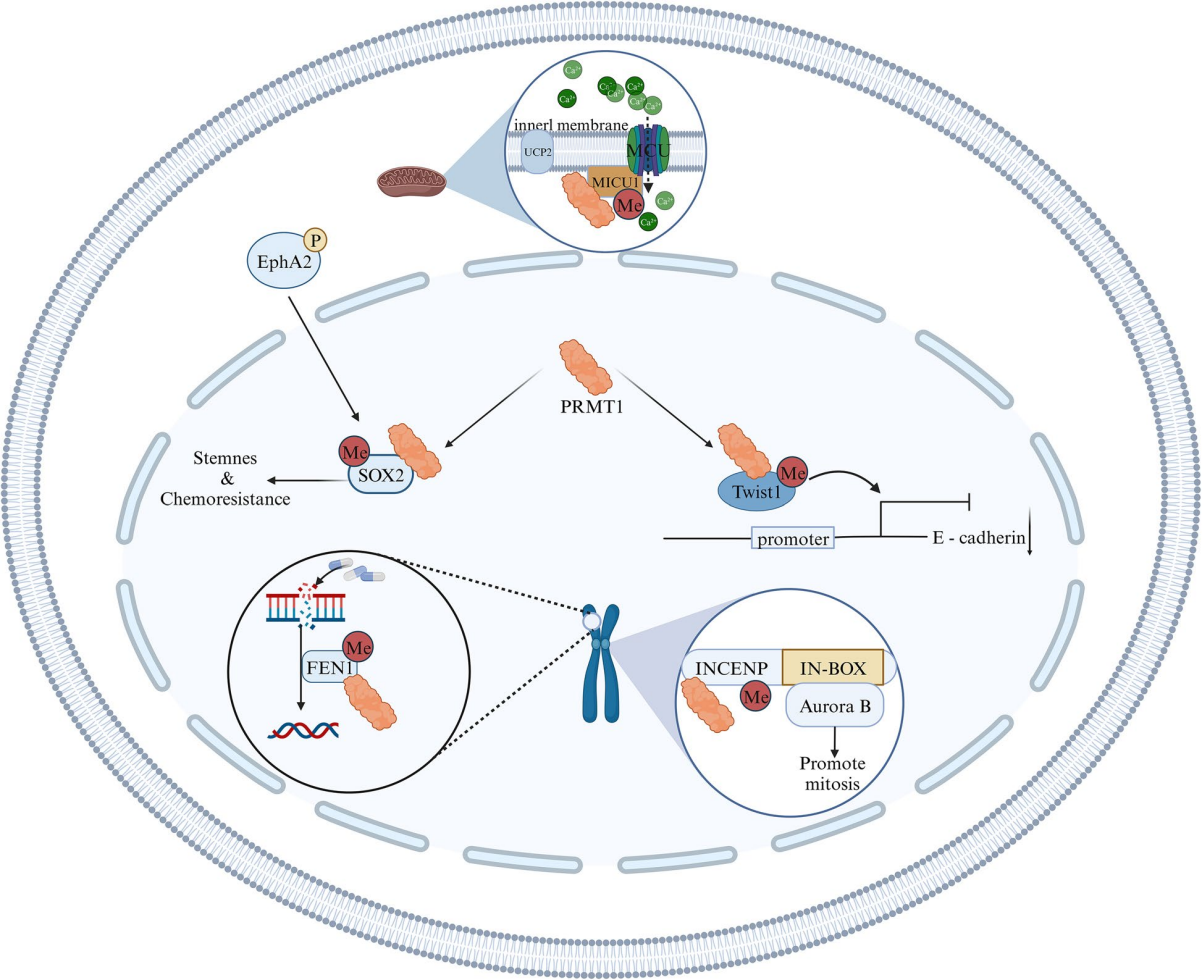
The mechanisms of action of PRMT1 in lung cancer. PRMT1 plays multiple roles in the pathogenesis of lung cancer, including involvement in DNA damage repair, influence on mitotic progression, regulation of transcriptional processes, and mitochondrial uptake of calcium ions

In the field of tumor cell metabolism, it is well-established that both glycolysis, which provides rapid energy production, and aerobic glycolysis, which offers efficient energy production, play a pivotal role in tumor progression [59, 60]. The proper concentration of calcium (Ca^2+^) within the mitochondria is essential for facilitating aerobic oxidation in tumor cells [61]. To counter mitochondrial Ca^2+^ overload, PRMT1 methylates MICU1, reducing its activity. Moreover, PRMT1 overexpression, and UCP2 collectively normalize mitochondrial Ca^2+^ uptake, ensuring oxidative phosphorylation (OXPHOS), aligning with the observed elevated levels of mitochondrial respiration in lung carcinoma cells. By regulating mitochondrial Ca^2+^ uptake, PRMT1 plays a crucial role in sustaining the vitality and proliferation of tumor cells [62]. In addition to its involvement in mitochondrial regulation, PRMT1 exerts effects on other cellular processes relevant to cancer. PRMT1 methylates INCENP at arginine 887, a region binding to AURKB. This methylation is advantageous for the activation of AURKB, promoting normal cell division and growth in cancer cells [63]. Furthermore, PRMT1 has been found to methylate Twist1, a known E-cadherin repressor, at arginine 34. This methylation is crucial for actively repressing E-cadherin, unveiling a novel regulatory mechanism of EMT in non-small cell lung cancer (NSCLC) [64]. The upregulation of PRMT1 in lung cancer has also been associated with chemotherapeutic drug resistance. Specifically, PRMT1 regulates the expression of FEN1, a major component of the Base Excision Repair pathway critical for DNA repair ability and chemotherapeutic drug response [65, 66]. Additionally, PRMT1 methylation of SOX2 at arginine 43 has been linked to increased stemness and chemotherapy resistance in small cell lung cancer (SCLC) [67]. Collectively, these findings underscore the multifaceted role of PRMT1 in cancer development and progression, impacting metabolism, cell division, EMT, and chemotherapeutic response (Fig. 4). Targeting PRMT1 holds promise as a potential therapeutic strategy in lung cancer.

### Liver cancer

**Fig. 5:**
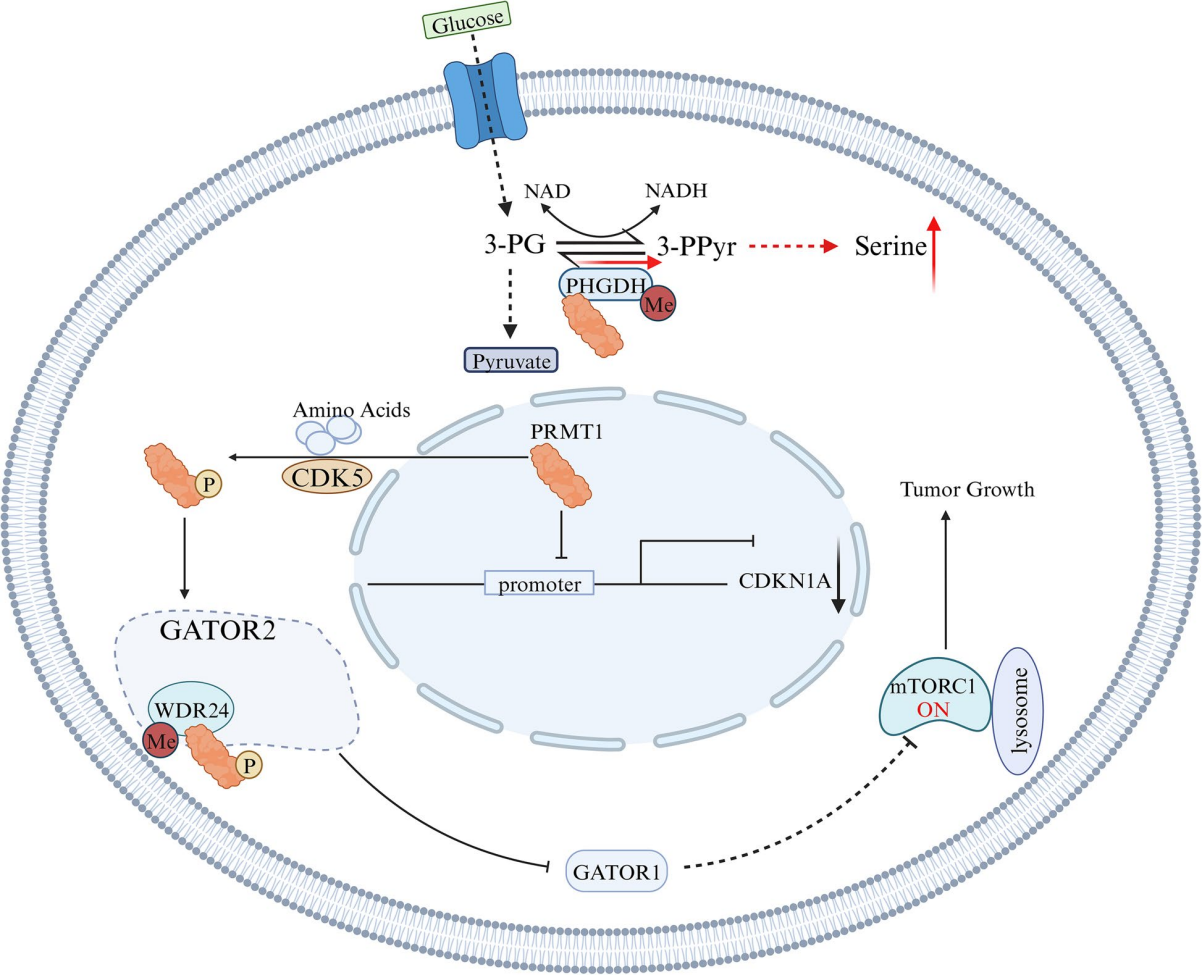
PRMT1 is involved in regulating transcription, oxidation, and signaling in liver cancer

PRMT1 has emerged as a significant player in liver cancer (Fig. 5), particularly hepatocellular carcinoma (HCC). Extensive research reveals significant overexpression of PRMT1 in HCC tissues compared to normal liver samples, as evidenced by the analysis of 50 normal liver samples and 371 HCC samples from The Cancer Genome Atlas (TCGA) database. Moreover, multiple patient cohorts have validated the prognostic value of PRMT1 in HCC, establishing it as a novel prognostic marker for assessing disease progression and patient outcomes [68]. This observation gains particular relevance given the underlying role of chronic inflammation in hepatocarcinogenesis, where hepatocytes, incited by chronic inflammation, transition from chronic hepatitis to cirrhosis, ultimately leading to liver cancer. Notably, myeloid-specific PRMT1 knockout mice exhibited reduced levels of IL-6 and suppressed STAT3 activity, protecting against alcohol-induced HCC development [69]. PRMT1 overexpression has been shown to activate the STAT3 signaling pathway by elevating STAT3 phosphorylation. Interestingly, inhibition of STAT3 phosphorylation using crypto tanshinone, a STAT3 inhibitor, reversed this process, highlighting PRMT1’s involvement in multiple regulatory mechanisms of the STAT3 pathway and suggesting its potential as a therapeutic target in HCC [70]. The deregulation of the mTORC1 pathway, linked to hepatocellular carcinoma cell proliferation, is influenced by GATOR2-mediated suppression of the GATOR1 protein. It has been reported that PRMT1 interacts with WDR24, a critical component of the GATOR2 complex, and enhances GATOR2’s inhibitory effect on GATOR1 by methylating it at arginine 329 [71]. Beyond these contributions, PRMT1 performs various cellular functions beyond its role as a transcriptional co-activator in epigenetic regulation, encompassing transcription, DNA repair, and signal transduction. The overexpression of PRMT1 in fresh HCC tissues has been associated with EMT, considered one of the hallmarks of cancer [72]. Mechanistically, activated Smad signaling due to PRMT1 activation fosters EMT through the TGF-β1/Smad pathway in hepatic carcinoma cells [73]. Notably, an inconsistency between HCC metabolomic and transcriptomic profiles has been observed, revealing that serine synthesis is activated despite downregulated mRNA and protein levels of PHGDH (phosphoglycerate dehydrogenase). Besides, PRMT1 plays a crucial role in augmenting PHGDH activity by methylating arginine 236, activating serine synthesis, enhancing the oxidative stress response, and promoting HCC growth [74]. Furthermore, a study by Ryu et al. unveiled the relationship between PRMT1 and CDKN1A (P21), whereby co-transfecting cells with siPRMT1 and siCDKN1A resulted in spheroid formation and restored cell growth, suggesting the involvement of CDKN1A downstream of PRMT1 in regulating tumor growth and formation in HCC [68]. In summary, elevated PRMT1 expression in HCC tissues, coupled with its prognostic value and involvement in various molecular pathways, underscores its potential as both a diagnostic marker and therapeutic target in liver cancer.

### Leukemia

**Fig. 6:**
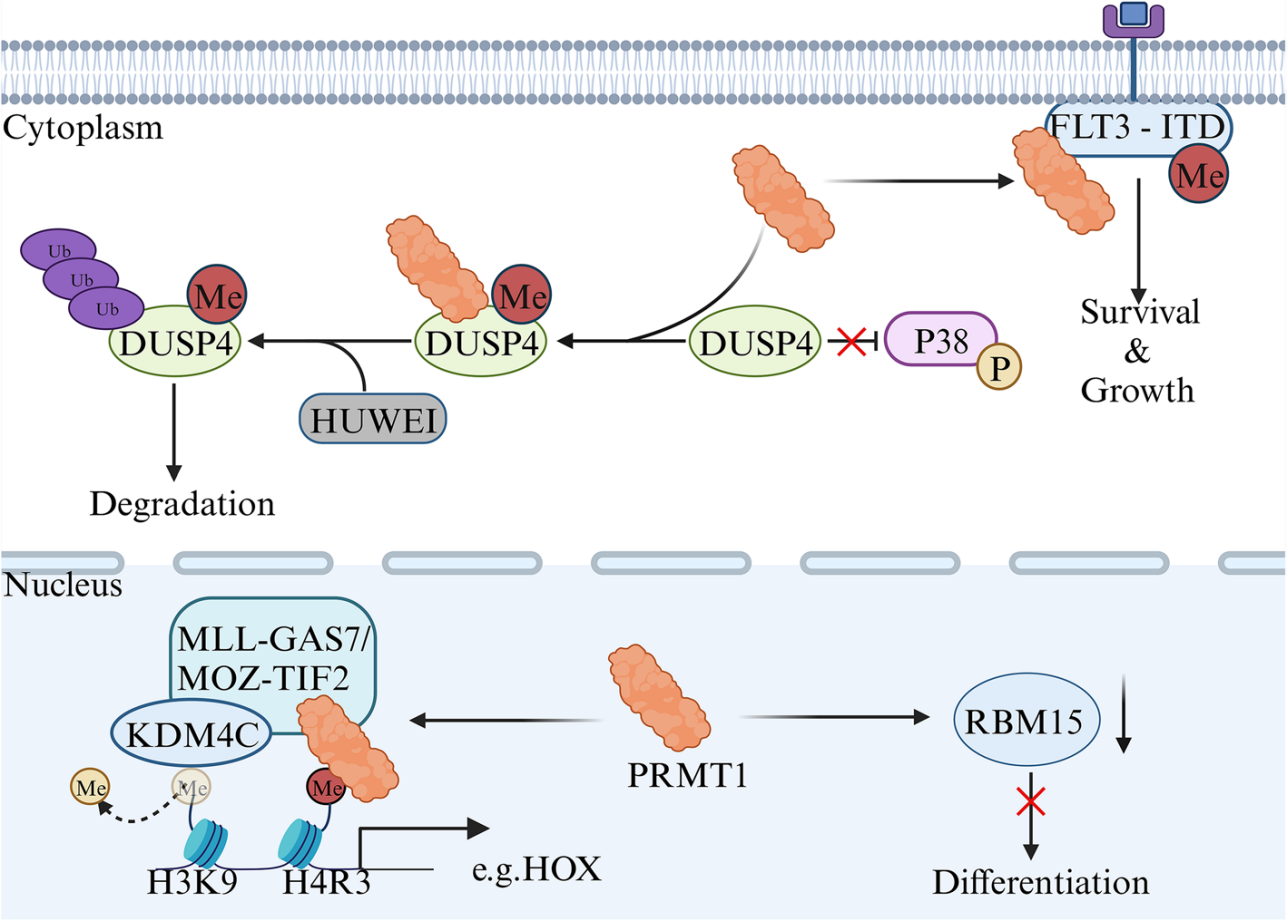
PRMT1 has a role in the regulation of transcription and signaling in leukemia

Optimal cell growth and differentiation heavily rely on the proper functioning of various epigenetic regulators, with PRMT1 being of significant importance. PRMT1 plays a crucial role in normal hematopoiesis, regulating myeloid differentiation and lymphocyte development [24, 75]. However, it has also been identified as a therapeutic target due to its involvement in hindering megakaryocyte differentiation in conditions like myelodysplastic syndrome (MDS) and megakaryocytic leukemia. PRMT1 hinders megakaryocyte terminal differentiation through the downregulation of RBM15 protein levels, affecting RNA splicing. RBM15, an RNA-binding protein, controls post-transcriptional processing [76, 77]. Additionally, PRMT1 is involved in the ubiquitination and degradation of DUSP4, a dual-specificity phosphatase in the P38 MAPK signaling pathway, leading to heightened P38 MAPK activity and the blockage of megakaryocyte differentiation [78]. Chromosomal translocations, such as MLL-GAS7 and MOZ-TIF2, produce abnormal oncogenic fusion proteins, highlighting PRMT1’s significance in leukemia pathogenesis. Studies indicate that MLL fusions and MOZ-TIF2-transformed cells recruit PRMT1 for mediating H4R3me2a. Besides, these cells also recruit KDM4C, a KDM, facilitating the removal of the H3K9me3 repressive mark. This synergistic effect enhances the transcription and transformation capability of these genes, contributing to acute myeloid leukemia (AML) development [79]. Moreover, methylation of PRMT1 at specific residues on FLT3-ITD protein amplifies its carcinogenic signal [80]. Inhibiting PRMT1 has promising potential to enhance the efficacy of FLT3-ITD^+^ AML kinase inhibitors, as well as in MLL-rearranged acute lymphoblastic leukemia [81]. PRMT1’s interaction with β-Catenin and HOXA9 in LSK-MLL-ENL cells plays a crucial role in mediating leukemia self-renewal, highlighting the significance of the HOXA9/PRMT1 molecular network beyond β-Catenin dependence [82]. Moreover, in the presence of GFI1, PRMT1 plays a pivotal role in the DNA damage repair pathway by methylating key molecules like MRE11 and 53BP1, contributing to efficient DNA damage repair and the development of lymphoid leukemia [83]. In summary, PRMT1 is a key epigenetic regulator in leukemia with diverse effects on cell growth and differentiation (Fig. 6). Understanding its mechanisms and interactions offers insights into potential therapeutic interventions for various hematological disorders.

### Colorectal cancer

**Fig. 7:**
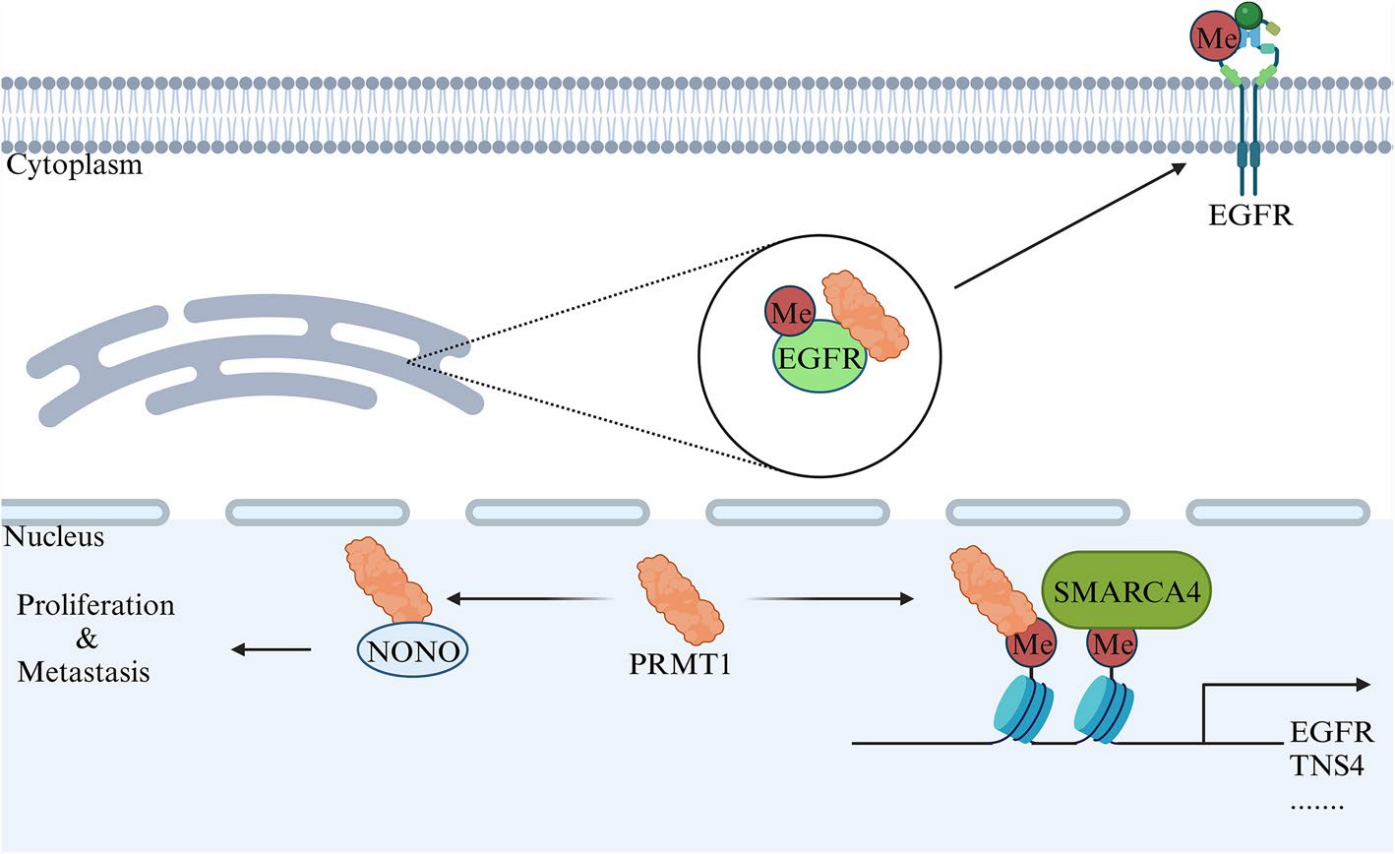
PRMT1 regulates transcription and signal transduction in colorectal cancer

The epidermal growth factor receptor (EGFR) family, an extensively researched receptor tyrosine kinase (RTK) system, plays crucial roles in normal development. Abnormal expression of EGFR is tightly correlated with various pathological conditions, particularly cancer [84, 85]. Cetuximab, a targeted therapy specifically designed for EGFR, has shown remarkable efficacy in treating colon cancer patients, resulting in improved prognosis and prolonged survival [86]. However, drug resistance remains a significant challenge for many patients. Recent studies have provided insights into the involvement of PRMT1 in the mechanisms of drug resistance within the EGFR signaling pathway. In a groundbreaking discovery by Liao et al., it was found that PRMT1 could methylate specific arginine residues, R198 and R200, located in the extracellular domain of EGFR. This arginine methylation by PRMT1, occurring in the endoplasmic reticulum (ER)/Golgi, subsequently leads to the transportation of methylated EGFR to the cell membrane. This facilitates receptor dimerization and signal activation, ultimately promoting cancer cell growth and conferring resistance to cetuximab treatment [87]. Additionally, PRMT1 enhances the transcriptional activity of EGFR, further fostering colon cancer cell proliferation, clonogenicity, and migration. Mechanistically, PRMT1 initiates asymmetric dimethylation of histone H4 on arginine 3, attracting SMARCA4. This process results in the formation of a complex that collaboratively activates the transcription of TNS4 and EGFR [88]. Furthermore, the SMARCA4^R1157W^ mutation increases SMARCA4 binding to PRMT1-mediated H4R3me2a, enhancing complex formation [89]. However, the presence of KRAS gene mutations presents another significant obstacle in treatment. In a related context, PRMT1-mediated methylation of NONO at position R251 has been shown to facilitate the growth and metastasis of colorectal cancer (CRC). Inhibiting PRMT1 demonstrated the potential to reduce NONO arginine methylation and suppress CRC progression, regardless of the mutation status of another crucial gene, *KRAS* [90]. These findings collectively suggest that inhibiting PRMT1 may hold significant therapeutic potential for CRC treatment, particularly in overcoming drug resistance mechanisms associated with EGFR-targeted therapies (Fig. 7).

### Esophageal cancer

**Fig. 8:**
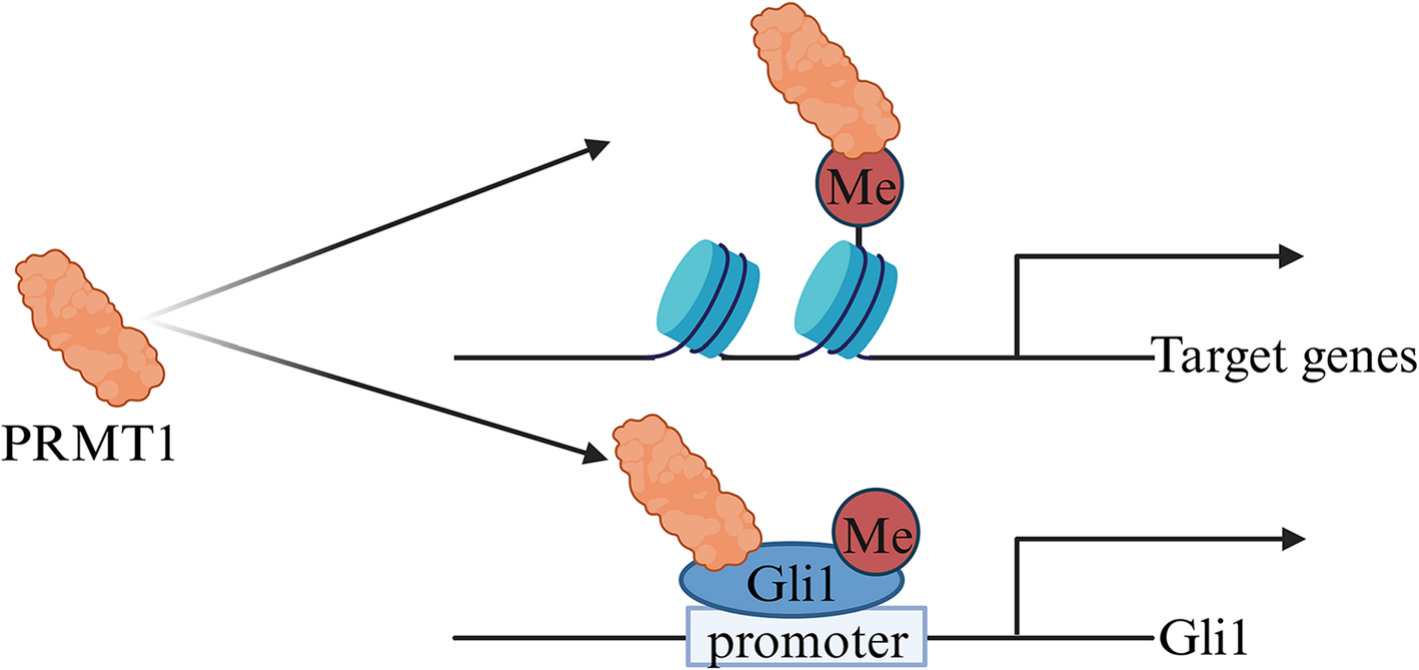
The mechanisms involved in transcriptional regulation by PRMT1 in esophageal cancer

Recent studies have unveiled that PRMT1, a protein arginine methyltransferase, is highly expressed in esophageal squamous cell carcinoma (ESCC) and is associated with poor prognosis. The role of PRMT1 in ESCC is twofold: firstly, it facilitates histone H4 methylation, thereby preserving the stem cell characteristics of ESCC [91]; secondly, it mediates Gli1 methylation, leading to an enhancement in its transcriptional activity [92]. The overexpression of PRMT1 triggers the activation of target genes downstream of the Hedgehog signaling pathway. Mechanistically, PRMT1 methylates the DNA-binding domain (DBD) of Gli1, known as glioma-associated oncogene homolog 1, thereby potentiating its transcriptional activity, promoting the growth and migration of esophageal squamous cell carcinoma cells. However, further validation is warranted to confirm the methylation site of Gli1, specifically R618 [92, 93]. Silencing PRMT1 has been found to significantly inhibit ESCC progression through the transcriptional activation mediated by histone H4R3me2a. Additionally, silencing PRMT1 reduces the self-renewal, tumorigenicity, and chemoresistance of OV6^+^ cells. RNA sequencing (RNA-seq) transcriptome analysis has further revealed that the overexpression of PRMT1 in ESCC cell lines activates the Wnt/β-catenin and Notch signaling pathways [91]. In conclusion, PRMT1, as a novel effector, plays a crucial role in promoting the stem cell characteristics of esophageal cancer while also maintaining tumor cell proliferation and migration (Fig. 8).

### Osteosarcoma

**Fig. 9:**
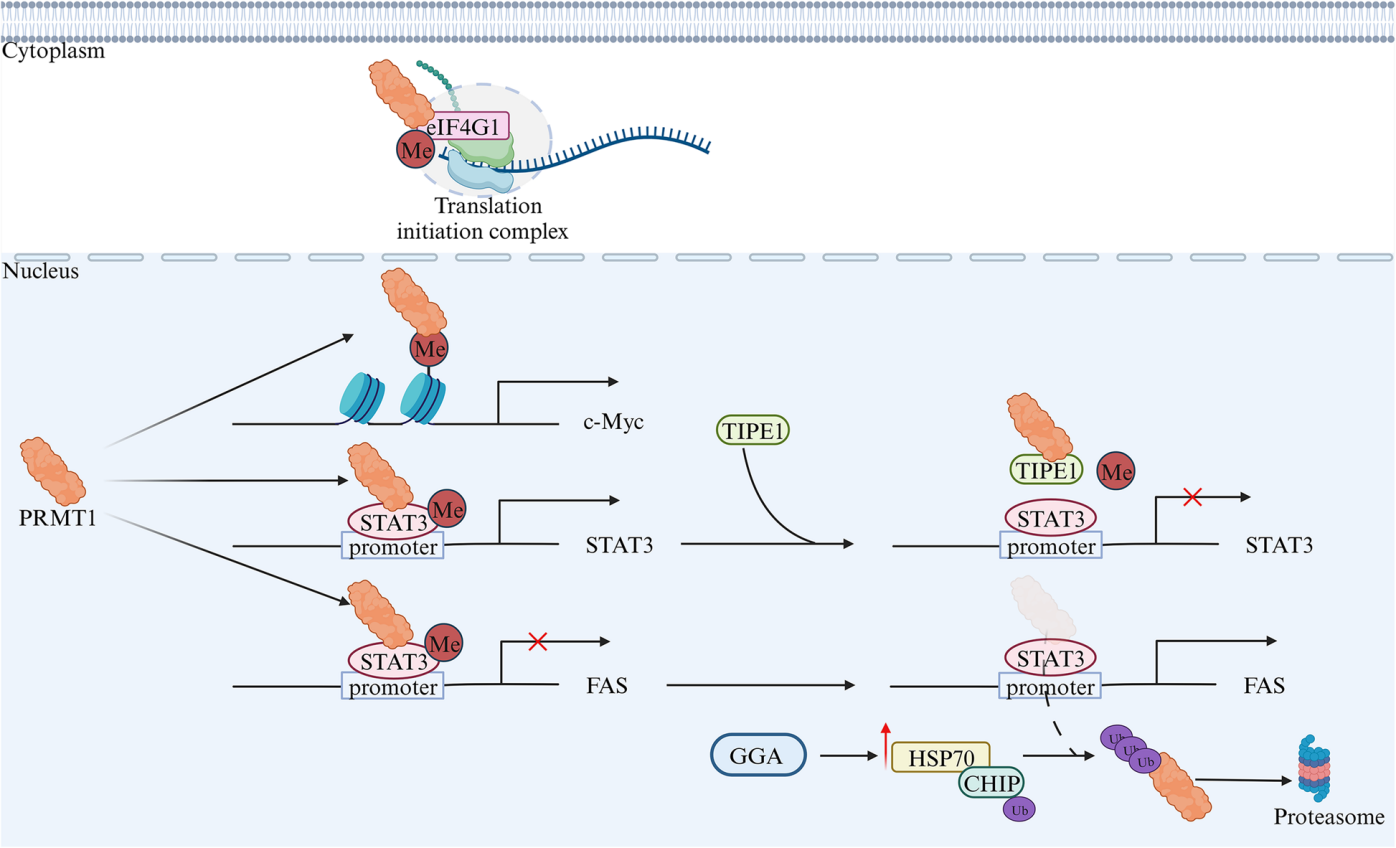
The role of PRMT1 in osteosarcoma. PRMT1 promotes the development and progression of osteosarcoma by regulating transcription and translation processes

Osteosarcoma is a type of bone cancer primarily observed in children and adolescents. The understanding of PRMT1’s mechanism in osteosarcoma tumorigenesis and the identification of exploitable vulnerabilities for targeted therapy are of utmost importance in the quest for effective treatment strategies (Fig. 9). Studies utilizing a mouse model with P53/RB deletion have highlighted the pivotal role of PRMT1 in osteosarcoma tumorigenesis. Deletion of PRMT1 in this context led to a significant increase in the lifespan of mice, emphasizing its significance in disease progression. PRMT1 exerts regulatory influence on the translation initiation complex in P53/RB-null cells by methylating eIF4G1 at positions R689 and/or R698, suggesting PRMT1’s involvement in promoting tumor initiation and maintenance through modulation of translation-associated genes at the translation level [94]. Li et al.’s study provides additional evidence of PRMT1’s role in promoting osteosarcoma at the translational level. It was found that PRMT1-mediated H4R3me2a could enhance c-Myc expression, activating the mTOR signaling pathway and promoting osteosarcoma progression [95]. Methylation of STAT3 at arginine 688 by PRMT1 promoted its transcriptional activation, contributing to osteosarcoma malignancy. TIPE1, by targeting the catalytic domain of PRMT1, inhibited its methyltransferase activity and suppressed osteosarcoma progression [96]. Moreover, it has been noted that the methylation and activation of STAT3 could prevent the transcription of FAS, thus hindering the apoptotic pathway initiated by FAS and supporting OS’s progression. The degradation of PRMT1 by GGA through the HSP70-CHIP-mediated proteasomal pathway was found to induce cell apoptosis triggered by FAS, suggesting potential therapeutic approaches for osteosarcoma [97]. In conclusion, understanding PRMT1’s role in osteosarcoma tumorigenesis, its interactions with translation-associated genes, and its involvement in the apoptosis pathway provides insights for developing targeted therapies against osteosarcoma.

## Prostate cancer

**Fig. 10:**
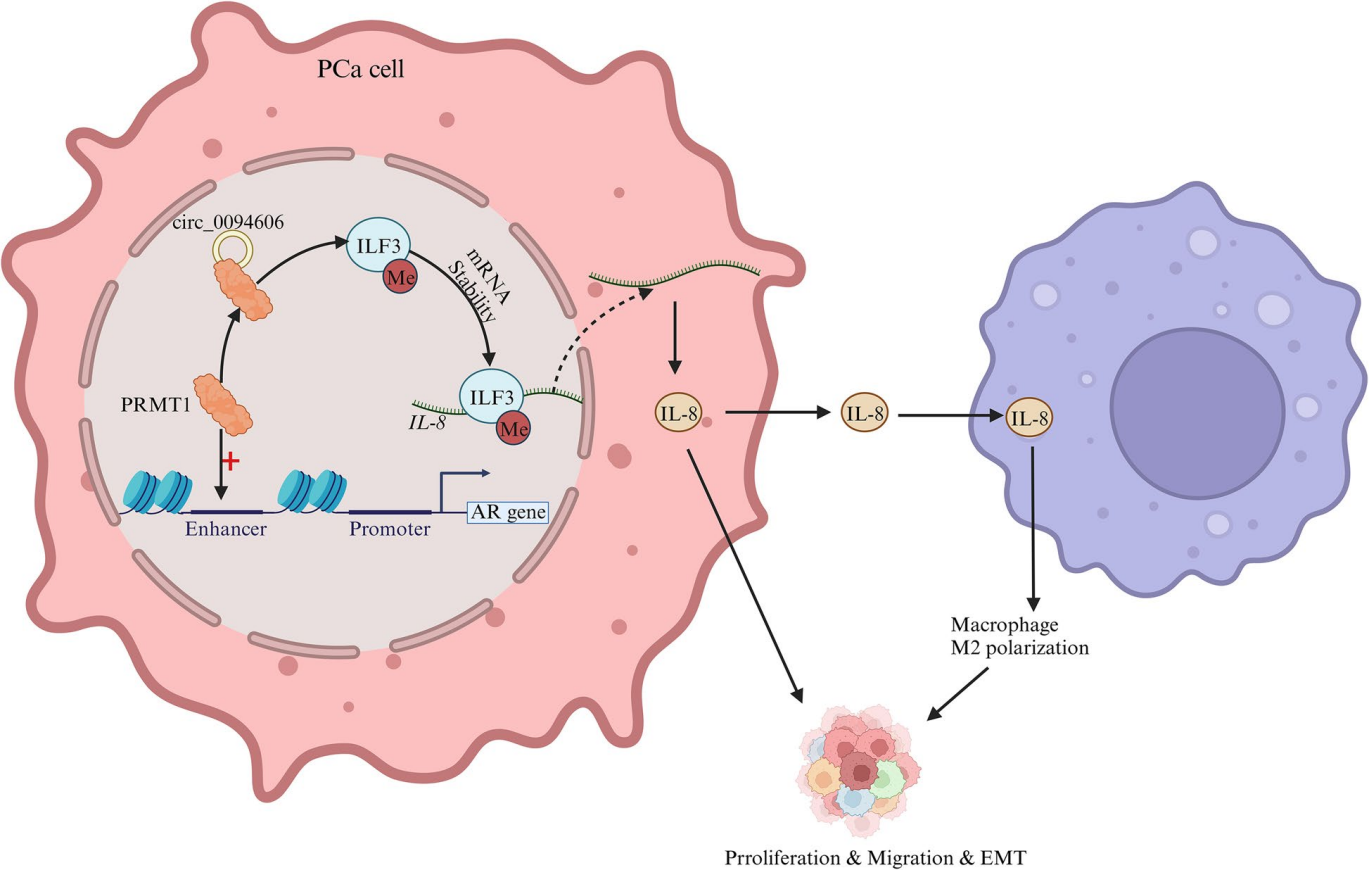
PRMT1 facilitates the transcription of the AR gene and translation of IL-8 in prostate cancer cells

Recent studies have highlighted the involvement of epigenetic networks, particularly PRMT1, in the progression of prostate cancer (PCa) (Fig. 10). The correlation between PRMT1 and CARM1 expression in PCa has been demonstrated, with PRMT1 overexpression associated with the EMT process [98]. PRMT1, when bound to hsa_circ_0094606, promotes M2 macrophage polarization by methylating ILF3 at R609, leading to the upregulation of IL-8 and enhanced proliferation, migration, and EMT processes in PCa cells [99]. A genome-scale CRISPR screen identified PRMT1 as a key mediator in the androgen receptor (AR) signaling pathway. Interestingly, it was found that PRMT1 could regulate the activity of AR enhancers, controlling the transcription and expression of the AR gene [100]. Targeting PRMT1 reduces the H4R3me2a modification at the AR locus, inhibiting AR-FL and AR-V7 transcription [101]. These findings offer insights into the resistance mechanisms of AR and AR-V7-driven castration-resistant prostate cancer, providing potential avenues for treatment.

### Ovarian cancer

**Fig. 11:**
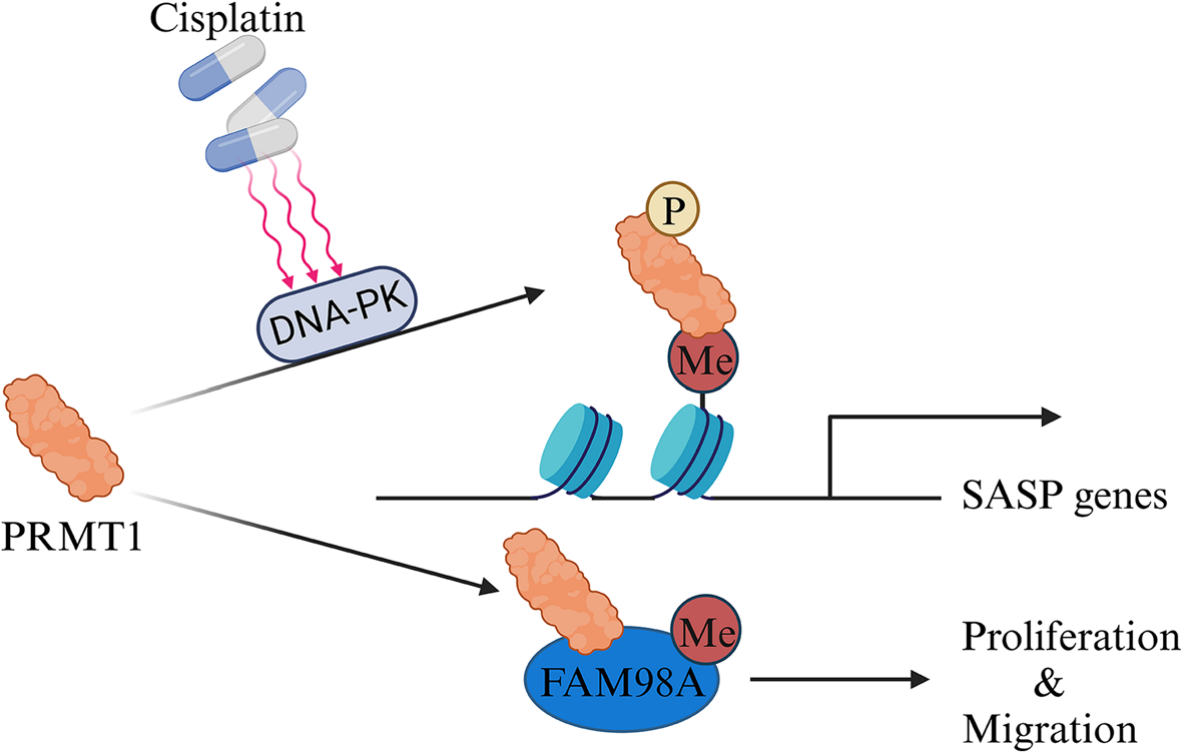
The mechanisms of PRMT1 in ovarian cancer involve promoting transcription and enhancing signal transduction

PRMT1 has also been identified as an oncogene in ovarian cancer (Fig. 11). It is typically expressed in various ovarian cancer cells, such as ES2 and SKOV3, and has been found to methylate FAM98A, a novel substrate of PRMT1. This methylation process promotes colony formation, in vivo proliferation, migration, and invasion of cancer cells [102]. PRMT1 also acts as a protective factor against cisplatin-induced apoptosis in ovarian cancer cells. When exposed to cisplatin, PRMT1 interacts with DNA-PK, undergoes phosphorylation at position 291, enhances its affinity for substrates, increases enzymatic activity, and accumulates in chromatin. The increased accumulation establishes a foundation for subsequent processes, facilitating the transcription of SASP (senescence-associated secretory phenotype) genes through the mediation of increased H4R3me2a levels. This modification plays a crucial role in maintaining the activation of the NF-κB -SASP axis. By inducing H4R3me2a, PRMT1 facilitates the expression of genes associated with the senescence-related secretory phenotype, blocking the sensitivity of cancer cells to CDDP [103]. Importantly, PRMT1 expression could serve as a predictive marker for sensitivity to platinum-based chemotherapy in patients with ovarian serous carcinoma [104].

### Gastric cancer

**Fig. 12:**
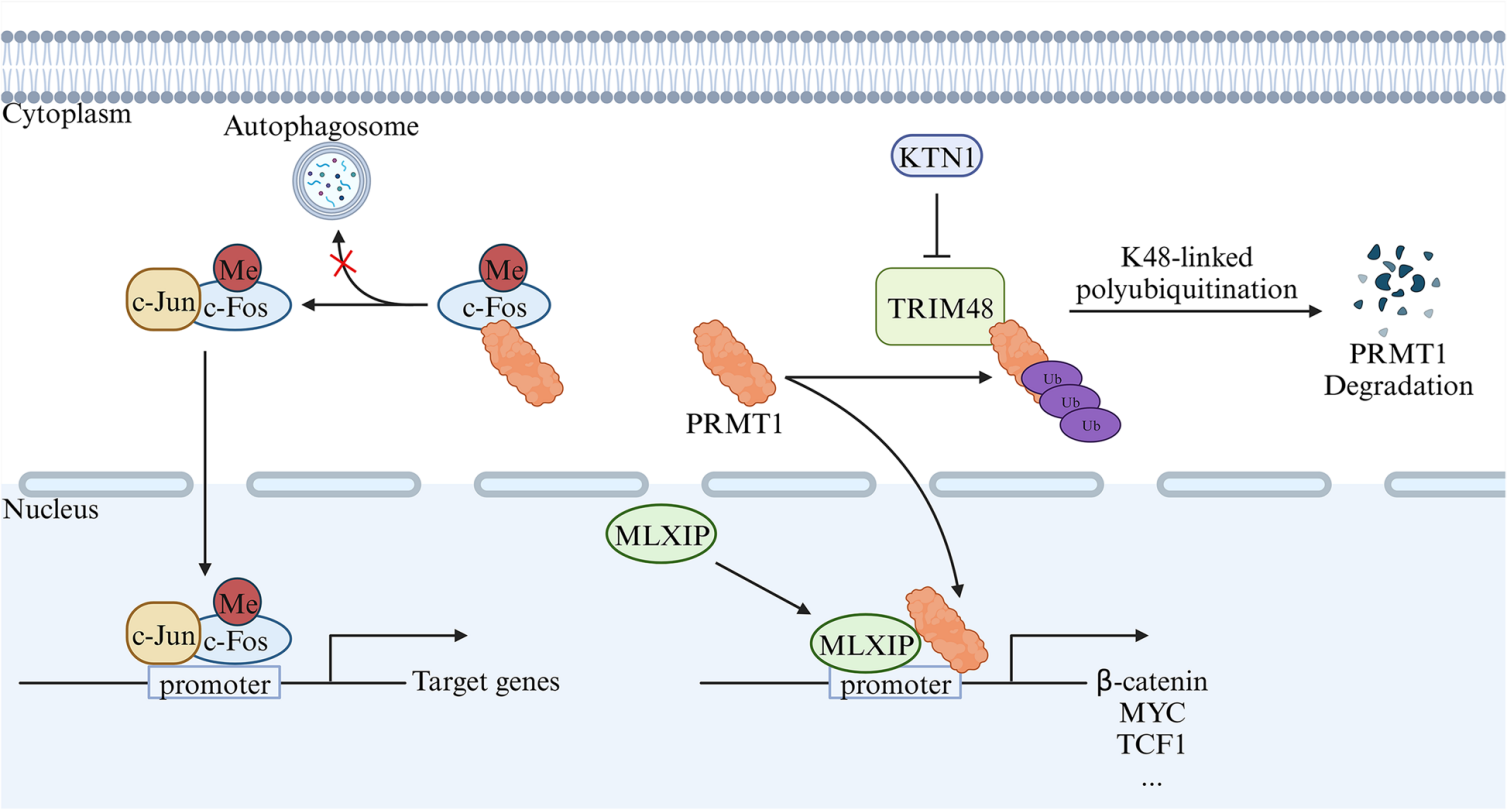
The mechanisms involved in transcriptional regulation by PRMT1 in gastric cancer

Bioinformatics analysis revealed significantly upregulated PRMT1 expression in gastric cancer tissue compared to normal tissue [105]. Wang et al. discovered the role of KTN1 in stabilizing PRMT1 protein expression by inhibiting K48-linked polyubiquitination through decreased TRIM48-PRMT1 interaction. Current evidence suggests that PRMT1 facilitates the recruitment of MLXIP to the promoter region of the β-catenin gene, triggering the transcription of the β-catenin signaling pathway. This activation promotes the proliferation, migration, and invasion of gastric cancer cells, leading to tumor growth and metastasis [106]. PRMT1 also plays a role in promoting the EMT process through the Hippo signaling pathway and enhances the migration and invasive ability of gastric cancer cells [107]. The direct interaction between c-Fos and PRMT1 synergistically enhances c-Fos-mediated AP-1 activity by methylating the R287 residue, protecting it from autophagy degradation. The increased stability of the c-Fos protein leads to increased AP-1 function, promoting gastric tumorigenesis [108]. These findings highlight PRMT1 as a potential therapeutic target in gastric cancer treatment (Fig. 12).

### Glioma

Glioma is recognized as the most aggressive type of brain tumor, and its highly infiltrative nature presents a significant challenge for achieving maximal safe resection. Temozolomide has become a crucial postoperative treatment for glioblastoma. Recent studies have elucidated the importance of arginine methylation, a post-translational protein modification, in the pathogenesis and progression of GBM. Dong et al. established the selective role of PRMT2 in facilitating the invasive phenotype of GBM [109]. PRMT5 regulates the proliferation and self-renewal of GBM neurospheres and is essential for the growth of GBM differentiated cells in serum [110]. Furthermore, PRMT6 was found to enhance mitotic activity [111] and cell cycle progression [112], thereby promoting tumor formation and progression in GBM models.

**Fig. 13:**
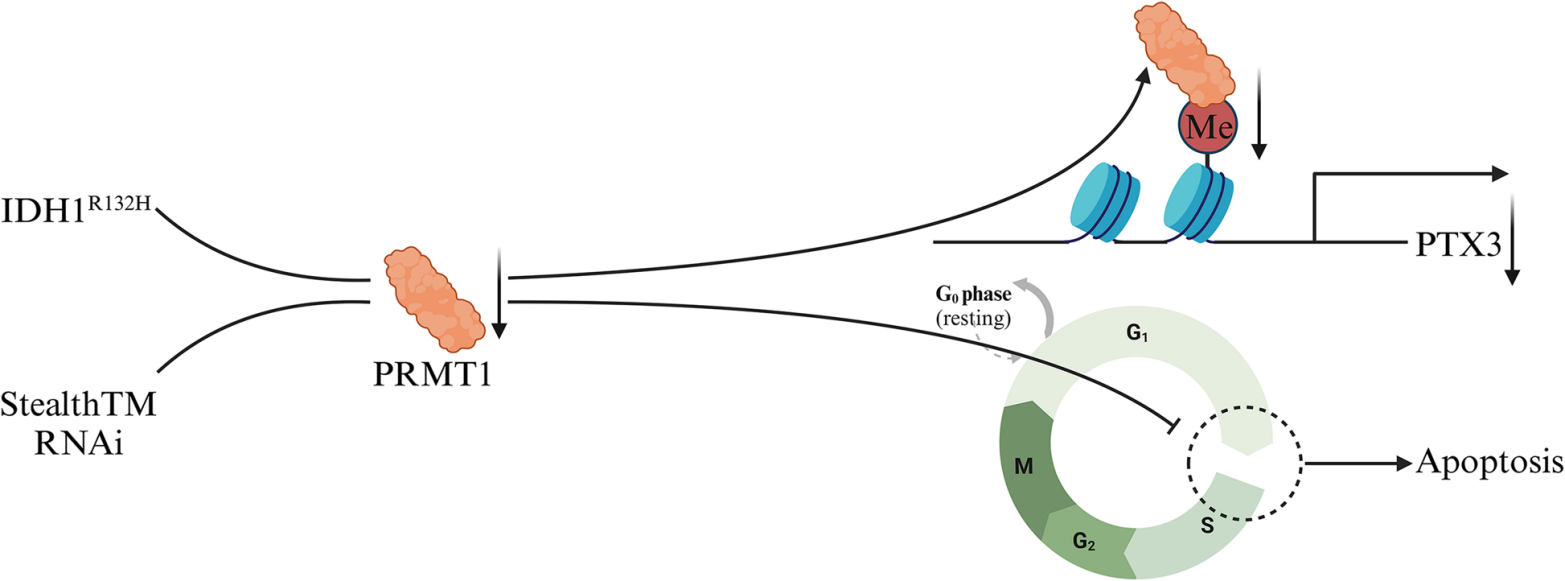
The mechanisms of action of PRMT1 in glioma. Reducing the expression of PRMT1 results in decreased transcription of PTX3 and induces apoptosis

PRMT1 has been found to be overexpressed in both human glioma tissue and cell lines compared to normal brain tissue (Fig. 13). Studies indicated that downregulating PRMT1 levels effectively arrested the cell cycle’s progression from the G1 to S phase in glioma cells exhibiting high PRMT1 levels, leading to the inhibition of proliferation and induction of apoptosis [113]. However, patients diagnosed with IDH1^R132H^ typically exhibit a noticeable decrease in PRMT1 expression. Previous studies have established that gliomas carrying the IDH1 mutant (IDH1^R132H^) exhibited antiproliferative characteristics due to hypermethylation of DNA and chromatin. Lathoria et al. recently identified additional mechanisms affecting cell death pathways in IDH1 mutant gliomas. Downregulated PRMT1 expression could hinder PTX3 transcription, resulting in escalated amalgamation between autophagosomes and lysosomes, along with an upsurge in ferritinophagy within glioma cells [114]. Ultimately, this leads to apoptosis in mutant glioma cells, holding the potential to improve patient prognosis.

### Others

In nasopharyngeal carcinoma, the expression of PRMT1 and RRM2 is significantly elevated in CNE-2 cells. PRMT1 was found to inhibit CNE-2 cell apoptosis by promoting RRM2 expression. The presence of PRMT1 in these cells could inhibit apoptosis by promoting the expression of RRM2 [115]. In renal cancer, inhibiting PRMT1 resulted in decreased deposition of H3R4me2a at the Lcn2 promoter, attenuating the transcription of Lcn2 and resulting in a decrease in its expression. Ultimately, the Akt-RB signaling pathway is mitigated. Inhibition of ccRCC cell proliferation is characterized by G1 phase cell cycle arrest, and it enhances the sensitivity of ccRCC cells to Sunitinib treatment [116].

### Tumor suppressive roles of PRMT1 in cancers

**Fig. 14:**
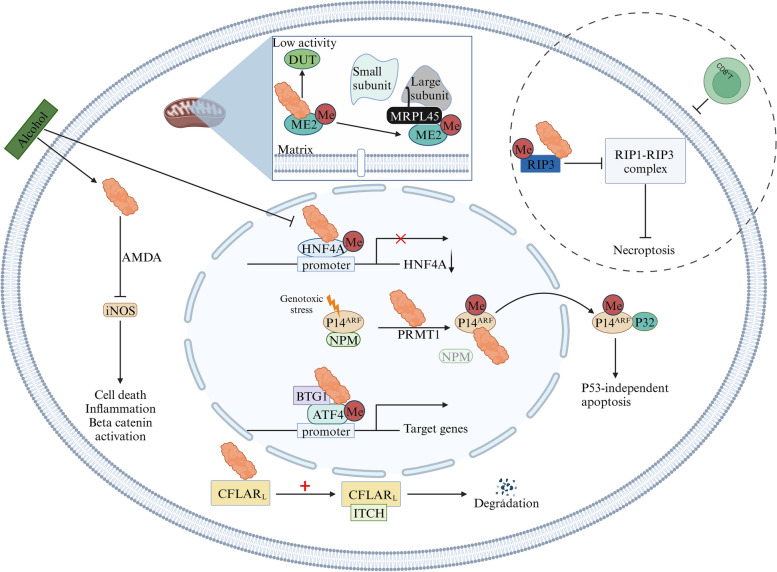
The mechanisms of cancer inhibition by PRMT1 in cancers

Many studies have shown that PRMT1 also acts as a tumor suppressor to prevent the occurrence of cancer (Fig. 14). In alcohol-induced liver cancer, alcohol inhibits the ability of PRMT1 to methylate arginine residues on the HNF4A promoter. This reduces HNF4A expression and promotes alcohol-related pathogenesis and proliferation of liver cancer cells [117]. The asymmetric dimethylarginine produced by PRMT1 inhibits the inducible nitric oxide synthase, reducing the production of nitrogen oxides and sulfur oxides, thereby weakening the oxidative stress and inflammatory response of the liver under the influence of alcohol, which in turn reduces cell death, inflammation, and the Wnt/β-Catenin signaling pathway activation, as well as tumor growth [118]. In addition, it has been revealed that PRMT1 could assist in the reversal of RIP3 necrotic colon cancer immune evasion. Mechanistically, PRMT1 methyltransferase is reportedly accountable for the methylation of RIP3, a central factor that accelerates necroptosis. This process of methylation obstructs the initiation of necroptosis by hindering the phosphorylation of RIP3, ultimately preventing the progression of tumor immune escape and necrotic colon cancer due to the initiation of necroptosis [119]. It has been found that PRMT1 could alleviate anemia symptoms in MDS patients [120]. Wang et al. elucidated the role of PRMT1 in mediating methylation of ME2 at the R67 site, which inhibited the conversion of ME2 monomers into dimers, resulting in decreased DUT activity, which mitigated thymidine and mtDNA generation. It has also been observed that the interaction between the MRPL45 and ME2 monomers could weaken the assembly of mitochondrial ribosomes and impair the production of mitochondrial proteins [121]. As a result, the progression of leukemia is significantly slowed. PRMT1 binds to ATF4 in a BTG1-dependent manner, and PRMT1 methylates ATF4, promoting transcription of some target genes of ATF4, leading to increased apoptosis in cancer cells [122]. In contrast to the function of PRMT5, PRMT1 overexpression has been found to modulate the protein level of CFLAR_L_, an antiapoptotic protein, through physical interactions. This interaction enhances the binding between CFLAR_L_ and the E3 ligase ITCH, resulting in changes in ubiquitination levels and ultimately leading to the degradation of CFLAR_L_. This degradation process increases the apoptosis of NSCLC cells [123]. Furthermore, under stress conditions, such as DNA damage in pancreatic cancer cells, PRMT1 has been observed to bind and methylate with P14^ARF^. This methylation caused ADMA at specific sites of the NLS/NoLS of P14^ARF^, namely R87/88/96/99. As a result, P14^ARF^ and NPM were separated in the nucleolus and redistributed to the nucleus and cytoplasm, which promoted non-dependent P53 apoptosis and improved the prognosis of patients [124]. It is worth noting that PRMT1 exhibits a dual role, as it not only promotes the EMT process but also inhibits the proliferation of gastric cancer cells [107]. Low expression of PRMT1 inhibits the accumulation of FOXO1 in the nucleus and reduces sensitivity to chemotherapy drugs, leading to relapse after adjuvant chemotherapy and a poor prognosis [125].

### PRMT1 inhibitors

A growing body of evidence from recent literature highlights the significance of PRMT1 in cancer research. Numerous studies have revealed that PRMT1 exhibits oncogene-like properties, playing a crucial role in regulating tumor cell proliferation, metastasis, and drug resistance. Consequently, it has been observed that PRMT1 is frequently overexpressed in tumor tissues, and its overexpression is often associated with poor prognosis. Given these findings, PRMT1 has emerged as a promising new research target for tumor therapy. As a result, there has been a surge of interest in the development of PRMT1 inhibitors as researchers strive to explore the potential of these inhibitors as cancer therapeutics (Table 1). The study of PRMT1 inhibitors has thus become a prominent area of focus in the quest for effective treatments against cancer.

**Table 1:**
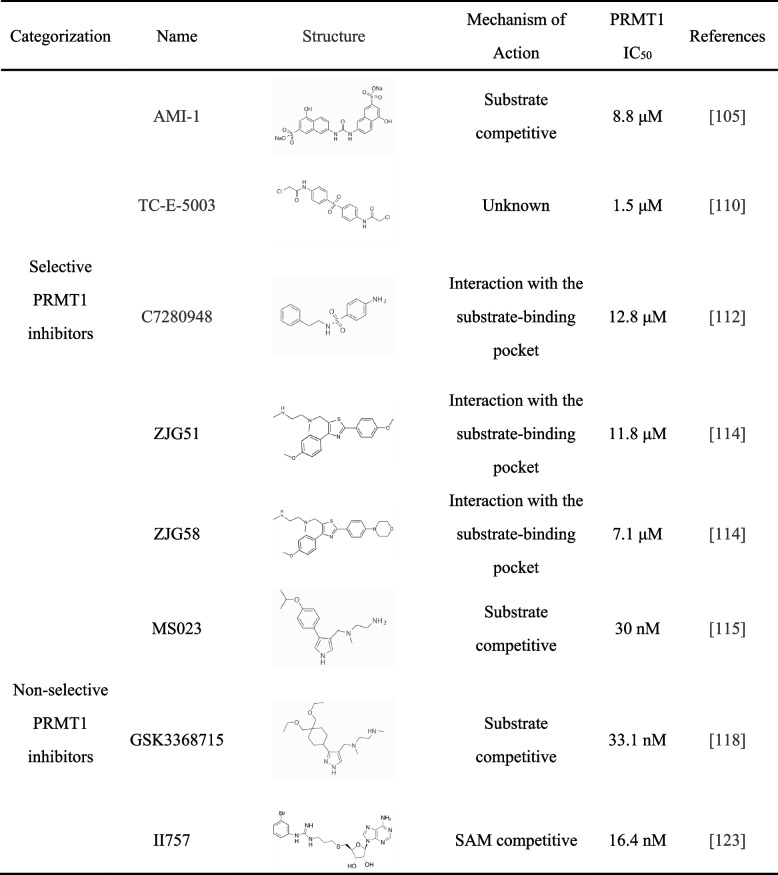
PRMT1 inhibitors

## Selective PRMT1 inhibitors

### AMI-1

AMI-1, also known as Disodium 7,7′-(carbonyldiimino) bis(4-hydroxynaphthalene-2-sulfonate), is the first discovered protein arginine methyltransferase inhibitor [126]. With a chemical formula of C_21_H_14_N_2_Na_2_O_9_S_2_, it also acts as an effective NADPH oxidase-derived superoxide scavenger [127]. AMI-1 exhibits some similarity to peptidyl arginine but functions as a SAM non-competitive inhibitor since AMI-1 may bind to the co-substrate binding site rather than interact with the substrate pocket [126, 128]. By blocking the entry of protein/peptide substrates, it selectively inhibits the interaction between the substrate (arginine residue) and the enzyme without competing with the AdoMet binding site [129]. AMI-1 has been widely studied and applied in various research experiments. Yin et al. demonstrated through Duolink PLA that NONO ADMA levels were significantly reduced in AMI-1-treated KM12 and HCT8 CRC cells compared to control cells. In xenograft models, mice treated with AMI-1 exhibited smaller tumors [90]. Following AMI-1 treatment, PRMT1-mediated proliferation of gastric cancer cells was inhibited, exhibiting a dose-dependent effect [106]. Due to the effective inhibition of arginine methyltransferase activity by AMI-1, further analogs of AMI-1 are currently being developed [130].

### Tc-e 5003

TC-E 5003 is a selective PRMT1 inhibitor, also known as N, N′-(sulfonyl-4,1-phenylene) bis(2-chloroacetamide). In the study by Kim et al., TC-E-5003 demonstrated promising anti-inflammatory effects by modulating the AP-1 and NF-κB signaling pathways induced by LPS, suggesting its potential as an anti-inflammatory compound [131]. In vitro experiments have shown that TC-E-5003 exhibits significant anti-tumor activity against lung cancer and breast cancer. Furthermore, by utilizing the INEI drug delivery system to deliver TC-E-5003-loaded INEI (40% NBCA) in an animal model, the average growth inhibition rate of xenografted human lung cancer cells was 68.23%, surpassing the inhibition rate achieved by TC-E-5003 alone (31.76%). This study further confirms the potential of the inhibitor TC-E-5003 as an anti-tumor drug and highlights INEI as an effective technique for enhancing anti-tumor effects [132].

### C-7280948

C-7280948 is a selective PRMT1 inhibitor that inhibits the activity of PRMT1 by binding to the substrate-binding pocket, with an IC_50_ value of 12.8 μM [133]. Studies have shown that treatment with C-7280948 can eliminate radiation resistance induced by PRMT1-mediated methylation of PKP2, making it a potential radiosensitizer in lung cancer [134]. Additionally, it can reduce the levels of ADMA in KM12 and HCT8 cells, thereby inhibiting the proliferation, migration, and invasion of colorectal cancer cells [90].

### ZJG51 and ZJG58

ZJG51 and ZJG58, selective inhibitors of type I PRMT, were designed and synthesized using molecular modeling to discover a new sub-binding pocket and occupy it by introducing a third substituent on the thiazole moiety. Molecular dynamics simulations revealed that ZJG51 - Pose 1 (where the ethylenediamine moiety is bound to the sublayer arginine binding site) emerged as the only stable binding site in the ZJG51 complex with PRMT1. Its additional aromatic substituent maintains its biological activity as a PRMT1 inhibitor by occupying the newly discovered sub-binding site. In comparison to ZJG58, ZJG51 exhibited potent inhibitory activity against all four tested tumor cells, with a remarkable effect against HeLa cells (IC_50_ = 9.43 ± 0.10 μM). Moreover, ZJG51 demonstrated efficacy in inducing apoptosis and inhibiting the migration of HeLa cells. Its mechanism of action may involve the activation of Caspase 9 and inhibition of EMT, both of which hold significant clinical implications for future cervical cancer treatment [135].

## Non-selective PRMT1 inhibitors

### MS023

The rational design and synthesis of MS023 involve the incorporation of the ethylenediamine side chain from EPZ020411 and CMPD-1, with the replacement of the pyrazole ring in EPZ020411 by a 1,2,3-triazole or pyrrole ring. MS023 is a selective inhibitor of type I PRMTs that exerts dose-dependent inhibition of PRMT1-mediated H4R3 methylation activity in MCF7 cells and suppresses the overexpression of PRMT6 in HEK293 cells [136]. MS023 can reportedly antagonize the methylation-promoting effect of PRMT1-mediated METTL14 on cell proliferation [18]. The combination of MS023 with PARP inhibitors could potentially serve as a novel therapeutic approach for MTAP-negative NSCLC and certain cancer cells that are resistant to PARP inhibitors [137]. In animal models treated with MS023, AML mice exhibited effective therapeutic outcomes [80], while significant inhibition of MM mice tumor growth was observed [138]. These findings indicate that MS023 has clinical value and the potential to be a valuable compound in the field of clinical oncology therapy.

### GSK3368715

GSK3368715 is a potent and reversible inhibitor of type I PRMTs that binds to the peptide site adjacent to the SAM pocket, making it a non-competitive peptide-mixed inhibitor of SAM [139]. As a monotherapy, GSK3368715 has shown increased sensitivity to ferroptosis in AML [140] and has the potential to enhance anti-tumor immune responses [141, 142]. Promising preclinical results and peripheral target engagement were observed at higher doses in a clinical study (TRIAL REGISTRATION NUMBER: NCT03666988). However, the occurrence of treatment-emergent adverse events (TEEs), variable target engagement at the tumor level, and limited clinical efficacy observed led to the premature termination of the trial [143].

### II757

The pan-inhibitor of arginine methyltransferases, II757, was carefully designed and synthesized by incorporating m-bromophenyl onto a guanidine moiety linked to a thioadenosine scaffold, inspired by the known inhibitor AH237. Through kinetic studies, it has been demonstrated that II757 exhibits competitive binding to the SAM binding site of PRMT1, thereby functioning as a potent SAM-competitive inhibitor of PRMT1 with an IC_50_ value of 16.4 nM. Importantly, II757 displays selectivity towards PRMTs over a range of other methyltransferases, making it a valuable tool and precursor for further exploration of PRMTs [144].

### Conclusions and perspectives

Methylation is a fundamental type of epigenetic regulation that involves the transfer of active methyl groups to target chemical substances under the catalysis of methyltransferases without altering the composition of the DNA sequence. Methylation can occur in DNA, RNA, and proteins, with arginine methylation being a frequently studied post-translational modification of proteins [145]. PRMT1 is the most prevalent arginine methyltransferase, and its expression is dysregulated in cancer. Numerous studies have demonstrated that PRMT1 is significantly expressed in different cancer types and is linked to tumor malignancy and prognosis. PRMT1 contributes to tumorigenesis by regulating transcription factors and signaling pathways, which also paves the way for the occurrence and development of tumors. Furthermore, the excessive expression of PRMT1 is linked to the conspicuous proliferation, invasion, and metastasis of tumorous cells, hence making it a crucial protein in cancer development.

A review of the literature reveals several key points about PRMT1. Firstly, PRMT1 exhibits different roles in different stages of tumor development, as demonstrated in alcohol-induced liver cancer. Under the stimulation of high-risk factors associated with alcohol consumption, PRMT1 functions normally to prevent tumor formation. However, varying degrees of alcohol intake can lead to different outcomes, such as decreased enzymatic activity of PRMT1. In alcohol-induced liver cancer cells, the normal biological function of PRMT1 may not be maintained, and instead, it may exhibit characteristics that promote cancer cell proliferation, invasion, and metastasis, thereby exerting a carcinogenic effect. The stimulatory and inhibitory effects of PRMT1 on growth may vary in different situations, while its ability to inhibit tumor growth depends on the stage of tumor development. Secondly, PRMT1 exhibits different mechanisms of action in regulating the same signaling pathway in different types of tumor cells. In the regulation of the EGFR signaling pathway, PRMT1 can act as a transcriptional regulator, promoting the expression of EGFR and enhancing signal transduction. It can also act as a methyltransferase, directly interacting with the EGFR molecule, leading to methylation of arginine residues in the extracellular domain of EGFR, thereby affecting the EGFR signaling pathway. Thirdly, the anticancer effect of PRMT1 is undeniable. Studies have found that compared to apoptosis, necroptosis is immunogenic, indicating that necroptosis in tumor cells can potentially be used as a therapeutic method by activating the immune system [146]. However, in necrotic colorectal cancer, a unique subtype of colon cancer, necroptosis can also provide a barrier for tumor cells, promoting tumor growth by creating an immune evasion microenvironment during the process of necroptosis. PRMT1 can reverse the immune evasion of tumor cells caused by necroptosis. Additionally, PRMT1 binds to ATF4 in a BTG1-dependent manner, methylating ATF4 and promoting the transcription of certain target genes of ATF4, leading to increased apoptosis of cancer cells. Under genotoxic stress conditions, PRMT1 promotes P53-independent cell apoptosis, improving the prognosis of pancreatic cancer patients, suggesting that PRMT1 also plays different mechanisms in inhibiting tumors.

Given the role of PRMT1 in tumors, it is imperative to further investigate the development and utilization of PRMT1 inhibitors. Table 1 presents the PRMT1 inhibitors that have been developed thus far, showcasing their ability to inhibit tumor cell proliferation and invasion, among other effects, underscoring their anti-tumor activity. Notably, GSK3368715 underwent a clinical trial that was prematurely terminated due to the occurrence of a TEE, variable engagement with the tumor target level, and limited clinical effectiveness. Additionally, GGA [97], spermine [101], and sodium propionate [147] have demonstrated inhibition of PRMT1. Therefore, it is crucial to explore more selective PRMT1-targeted inhibitors that have fewer side effects and improved efficacy. Furthermore, it is equally important to consider new therapeutic avenues of research, such as prime editing [148], proteolysis targeting chimera (PROTAC) [149], and CAR-T cell therapy [150].

It is well-established that PRMT1 plays a crucial role in regulating gene transcription, protein expression, and tumor cell differentiation, which contribute to the proliferative and metastatic behaviors exhibited by tumors. Additionally, PRMT1 is involved in promoting DNA damage repair, leading to the acquisition of antiapoptotic and drug-resistant characteristics in tumor cells. The role of PRMT1 in regulating the tumor microenvironment has also been gradually recognized, although its potential involvement in promoting tumor angiogenesis remains an area of unknown research. Furthermore, the exact mechanisms by which PRMT1 functions in various signaling pathways, such as the Hedgehog and Hippo signaling pathways, warrant further investigation.

**Fig. 15:**
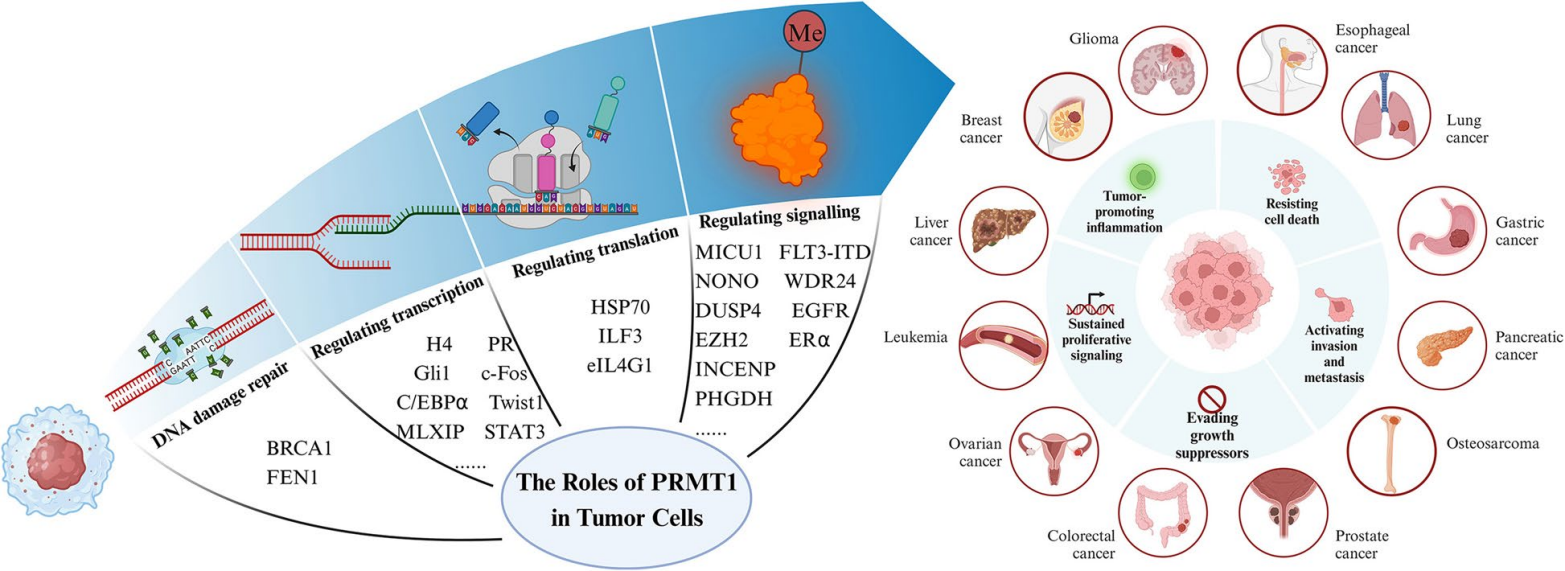
The roles of PRMT1 in tumor cells

Herein, we provide a systematic introduction to the role of PRMT1 in tumors (Fig. 15). The dysregulated expression of PRMT1 in tumors underscores its considerable impact. Additionally, emerging research indicates that PRMT1 is implicated in inflammatory diseases [151], cardiovascular diseases [152], neurological disorders [38], and immune disorders [153], emphasizing its pivotal role in the development of various diseases. As a highly valuable arginine methyltransferase, PRMT1 holds substantial research potential, providing crucial insights into the progression of diverse diseases. In summary, while PRMT1 has demonstrated a significant impact on tumor development, a comprehensive understanding of its role and potential therapeutic applications remains a work in progress.

## Data Availability

Not applicable.

## Abbreviations

PRMTs: Protein arginine methyltransferases
MMA: Monomethylarginine
ADMA: Asymmetric dimethylarginine
SDMA: Symmetric dimethylarginine
SAM: S-Adenosyl methionine
SAH: S-adenosylhomocysteine
GAR: Glycine- and arginine-rich
PGM: Post-translational glycine methylation
RGG: Arg-Gly-Gly
53BP1: P53-binding protein 1
MRE11: Meiotic recombination 11 homolog A
γ-IR: Gamma ionizing radiation
METTL14: Methyltransferase like 14
PR: Progesterone receptor
EMT: Epithelial-mesenchymal transition
BCL2: B-cell lymphoma 2
PI3K: Phosphatidylinositide 3-kinases
AKT (PKB): Protein kinase B
TAMs: Tumor-associated macrophages
IL-6: Interleukin-6
EZH2: Enhancer of zeste homolog 2
PRC2: Polycomb repressive complex 2
C/EBPα: CCAAT/enhancer binding proteins α
SG: Stress granule
PARP: Poly (ADP-ribose) polymerase
HP1γ: Heterochromatin protein 1γ
HDAC1/2: Histone deacetylases 1 and 2
LSD1: Lysine-specific demethylase 1
CDK1: Cyclin-dependent kinase 1
AMPK: Adenosine monophosphate-activated protein kinase
TRAF6: TNF receptor-associated factor 6
SUZ12: Suppressor of zeste 12 protein homolog
DAB2IP: Disabled homolog 2-interacting protein
CSTA: Cystatin A
P16: Cyclin-dependent kinase inhibitor 2A
P21: Cyclin-dependent kinase inhibitor 1A
ZEB1: Zinc finger E-Box binding homeobox 1
EGFR: Epidermal growth factor receptor
HDAC3: Histone deacetylase 3
circTBC1D14: Circular RNA TBC1D14
P53: Tumor protein p53
BRCA1: Breast cancer type 1 susceptibility protein
BARD1: BRCA1-associated RING domain protein 1
c-Myc: Cellular myelocytomatosis oncogene
ERα: Estrogen receptor alpha
IGF-1: Insulin-like growth factor 1
IGF-1R: Insulin-like growth factor 1 receptor
IRS1: Insulin receptor substrate 1
Shc: Src homology 2 domain-containing transforming protein
Grb2: Growth factor receptor-bound protein 2
ERK: Extracellular signal-regulated kinase
MICU1: Mitochondrial Ca2^+^ uptake 1
UCP2: Uncoupling protein 2
OXPHOS: Oxidative phosphorylation
INCENP: Inner centromere protein
AURKB: Aurora kinase B
SCLC: Small cell lung cancer
FEN1: Flap endonuclease 1
NSCLC: Non-small cell lung cancer
HCC: Hepatocellular carcinoma
TCGA: The Cancer Genome Atlas
DDR: DNA damage response
TCF: T-cell factor
CTNNB1: Catenin beta 1
Gli1: Glioma-associated oncogene 1
HSP70: Heat shock protein 70
EphA2: Erythropoietin-producing hepatocellular A2
SOX2: SRY-Box transcription factor 2
STAT3: Signal transducer and activator of transcription 3
mTORC1: Mammalian target of rapamycin complex 1
GATOR1: GTPase-activating protein (GAP) activity towards rags 1
WDR24: WD repeat-containing protein 24
TGF-β1: Transforming growth factor beta 1
CDKN1A: Cyclin-dependent kinase inhibitor 1A
MDS: Myelodysplastic syndrome
RBM15: RNA-binding motif protein 15
DUSP4: Dual-specificity phosphatase 4
P38 MAPK: p38 mitogen-activated protein kinase
MLL-GAS7: Mixed-lineage leukemia-growth arrest-specific 7
MOZ-TIF2: Monocytic leukemia zinc finger protein- transcriptional intermediary factor 2
H4R3me2a: Asymmetric dimethylation of histone H4 on arginine 3
H3K9me3: Trimethylation of histone H3 on lysine 9
KDM4C: Lysine-specific demethylase 4C
AML: Acute myeloid leukemia
MLL: Mixed-lineage leukemia
FLT3: FMS-like tyrosine kinase 3
ITD: Internal tandem duplications
HOXA9: Homeobox A9
LSK: Lin^−^ Sca-1^+^ c-kit^+^
ENL: Eleven-nineteen-leukemia;
GFI1: Growth factor independence 1
SMARCA4: SWI/SNF related matrix-associated actin-dependent regulator of chromatin subfamily A member 4
TNS4: Tensin-4
KRAS: Kirsten rat sarcoma viral oncogene homolog
NONO: Non-POU domain-containing octamer-binding protein
P53/RB: Tumor protein 53/retinoblastoma protein
eIF4G1: Eukaryotic translation initiation factor 4 gamma 1
TIPE1: Tumor necrosis factor alpha-induced protein 8-like 1
CHIP: Carboxyl terminus of HSP70-interacting protein
FAS: Tumor necrosis factor receptor superfamily member 6
hsa_circ_0094606: *Homo sapiens* circular RNA 0094606
CRISPR: Clustered regularly interspaced short palindromic repeats
ILF3: Interleukin-enhanced binding factor 3
FAM98A: Family with sequence similarity 98 member A
DNA-PK: DNA-dependent protein kinase
NF-κB: Nuclear factor kappa-light-chain-enhancer of activated B cells
CDDP: Cisplatin
KTN1: Kinectin 1
TRIM48: Tripartite motif-containing 48
MLXIP: MLX-interacting protein
AP-1: Activator protein 1
GBM: Glioblastoma
IDH1: Isocitrate dehydrogenase (NADP^+^) 1
PTX3: Pentraxin 3
RRM2: Ribonucleotide reductase regulatory subunit M2
Lcn2: Lipocalin 2
ccRCC: Clear cell renal cell carcinoma
HNF4A: Hepatocyte nuclear factor 4 alpha
RIP3: Receptor interacting protein kinase 3
ME2: Malic enzyme 2
DUT: Deoxyuridine 5′-triphosphate nucleotidohydrolase
mtDNA: Mitochondrial DNA
MRPL45: Mitochondrial ribosomal protein L45
BTG1: B-cell translocation gene 1
ATF4: Activating transcription factor 4
CFLAR_L_: CASP8 and FADD-like apoptosis regulator
P14^ARF^: p14 Alternate Reading Frame (ARF) protein
NLS/NoLS: Nuclear localization signal/nucleolar localization signal
FOXO1: Forkhead box O1
NADPH: Nicotinamide adenine dinucleotide phosphate
CRC: Colorectal cancer
INEI: Injectable NBCA ethyl oleate implant
NBCA: N-butyl-2-cyanoacrylate
MTAP: Methylthioadenosine phosphorylase
MM: Multiple myeloma
CAR-T: Chimeric antigen receptor T-cell therapy
RTK: Receptor tyrosine kinase
ESCC: Esophageal squamous cell carcinoma
DBD: DNA binding domain
LPS: Lipopolysaccharide
PKP2: Plakophilin 2
GGA: Geranylgeranylacetone
TEEs: Treatment-emergent adverse events
NPM: Nucleophosmin
PROTAC: Proteolysis targeting chimera
3-PG: 3-phosphoglycerate
3-PPyr: 3-phosphopyruvate
HUWEI: HECT, UBA and WWE domain containing E3 ubiquitin protein ligase 1
ITCH: Itchy E3 ubiquitin protein ligase
iNOS: Inducible nitric oxide synthase.
